# PP2C phosphatases Ptc1 and Ptc2 dephosphorylate PGK1 to regulate autophagy and aflatoxin synthesis in the pathogenic fungus *Aspergillus flavus*

**DOI:** 10.1128/mbio.00977-23

**Published:** 2023-09-27

**Authors:** Zhuo Zhu, Mingkun Yang, Guang Yang, Bei Zhang, Xiaohong Cao, Jun Yuan, Feng Ge, Shihua Wang

**Affiliations:** 1 State Key Laboratory of Ecological Pest Control for Fujian and Taiwan Crops, Key Laboratory of Biopesticide and Chemical Biology of Education Ministry, Key Laboratory of Pathogenic Fungi, School of Life Sciences, Fujian Agriculture and Forestry University, Fuzhou, China; 2 Mycotoxins of Fujian Province, School of Life Sciences, Fujian Agriculture and Forestry University, Fuzhou, China; 3 State Key Laboratory of Freshwater Ecology and Biotechnology, Institute of Hydrobiology, Chinese Academy of Sciences, Wuhan, China; Duke University School of Medicine, Durham, North Carolina, USA

**Keywords:** PP2C phosphatase, phosphoglycerate kinase 1, IP-MS, *Aspergillus flavus*, aflatoxin

## Abstract

Reversible phosphorylation by protein kinases and phosphatases plays a central role in regulating cellular processes. However, knowledge of the functions of protein phosphatase 2C (PP2C) S/T phosphatases in *Aspergillus flavus* has been unreported until now. Here, we have identified seven members of the PP2C family of protein phosphatases in *A. flavus*. Evolutionary and functional analyses indicated that two redundant PP2C phosphatases, Ptc1 and Ptc2, are highly conserved and regulate conidia development, aflatoxin synthesis, seed infection, and autophagic vesicle formation. The cytoplasmic proteins Ptc1 and Ptc2 exhibit nuclear infiltration after DNA damage-induced autophagy. Their degradation is closely linked to autophagy induction. The Asp residue coordinated with Mg^2+^ is critical for phosphatase Ptc1 and Ptc2 activity, thermal stability, and biofunction in *A. flavus*. An immunoprecipitation-mass spectrometry proteomic investigation indicated that 133 proteins co-interact with Ptc1 and Ptc2. Among these proteins, phosphoglycerate kinase 1 (PGK1) interacts with Ptc1 and Ptc2 and shows high levels of phosphorylation in Δ*ptc1*, Δ*ptc2*, and Δ*ptc1*/*ptc2* mutants. Furthermore, PGK1 S203 phosphorylation levels are associated with aflatoxin synthesis and autophagic vesicle formation. Overall, these findings contribute to our understanding of the roles and mechanisms of PP2C family phosphatases in *A. flavus* and highlight their importance in various cellular processes. Furthermore, it reveals a new regulation model in *A. flavus*, where Ptc1 and Ptc2 activate autophagy and aflatoxin synthesis by regulating PGK1.

**IMPORTANCE:**

*Aspergillus flavus* is a model filamentous fungus that can produce aflatoxins when it infects agricultural crops. This study evaluated the protein phosphatase 2C (PP2C) family as a potential drug target with important physiological functions and pathological significance in *A. flavus*. We found that two redundant PP2C phosphatases, Ptc1 and Ptc2, regulate conidia development, aflatoxin synthesis, autophagic vesicle formation, and seed infection. The target protein phosphoglycerate kinase 1 (PGK1) that interacts with Ptc1 and Ptc2 is essential to regulate metabolism and the autophagy process. Furthermore, Ptc1 and Ptc2 regulate the phosphorylation level of PGK1 S203, which is important for influencing aflatoxin synthesis. Our results provide a potential target for interdicting the toxicity of *A. flavus*.

## INTRODUCTION

The reversible phosphorylation of proteins by protein kinases and phosphatases regulates fungal cellular processes, including metabolism, transcription, and the cell cycle in fungi (1, 2). As a transient and reversible post-translational modification (PTM), substrate protein phosphorylation allows cells to change the conformation, activity, and interactions of the target proteins (3). PTM is a major molecular switch that maintains strict spatiotemporal control over target proteins at specific sites and is involved in almost every cellular process in the organelles (4, 5). Changes in protein conformation and interactions mediate subcellular localization and the turnover of its targets and reach the targeted functional region (5). For example, in this way, metabolic enzymes, such as phosphoglycerate kinase 1 (PGK1), exhibit subcellular translocation to participate in biological processes after being reversibly phosphorylated (6, 7). The mitochondrial translocation of PGK1 is mediated by PGK1 S203 phosphorylation and subsequent peptidyl-prolyl cis/trans isomerase NIMA-interacting1 (PIN1)-mediated cis-trans isomerization, and acts as a protein kinase in coordinating glycolysis and the tricarboxylic acid (TCA) cycle (6, 8). Therefore, the correct intracellular distribution of reversibly phosphorylated proteins is critical for the function of eukaryotic cells (9). Generally, protein phosphorylation occurs on two of the hydroxyl-containing amino acids, serine and threonine, although it can also occur on tyrosine (1). Protein phosphatase 2C (PP2C), a well-conserved serine/threonine (S/T) phosphatase, is critical in S/T protein dephosphorylation and is involved in regulating multiple biological processes (10). The dephosphorylation activity of PP2C phosphatases, which are members of the metal-dependent protein phosphatase (PPM) family, depends on various metal ions, such as Mg^2+^, Mn^2+^, or Ca^2+^ (1, 11, 12). These ions are often located at the enzyme center to mediate the nucleophilic attack on the phosphorous atom and increase the substrate specificity of dephosphorylation, thus they contribute to fine-tuning of the process.

In *Saccharomyces cerevisiae*, seven members of the PP2C subfamily (PTC1–PTC7) have been extensively explored and are involved in various biological pathways, including the Slt2-mediated cell wall integrity, high osmolarity glycerol (HOG), target of rapamycin (TOR), and cell wall integrity (CWI) pathways (13 – 16). Ptc1 and Ptc6 contribute to maintaining Slt2 in the dephosphorylated state and vacuole morphogenesis under cell wall stress (15). Ptc1, Ptc2, and Ptc3 inactivate Hog1 to negatively regulate the HOG pathway during adaptation to osmotic stress (14, 17). In addition, Ptc1 negatively regulates the CWI pathway by inactivating the mitogen-activated protein kinase 1(MPK1) cascade and positively regulates the pheromone response mitogen-activated protein kinase (MAPK) FUS3 cascade (18, 19). Atg1-Atg13-Atg17, the central complex in the TOR pathway, is frequently phosphorylated and repressed by protein kinase A and TORC1-dependent phosphorylation (20 – 22). Recently, Ptc2 and Ptc3 were revealed to be positive regulators of macroautophagy through the dephosphorylation of Atg13 and Atg1, which may be involved in autophagy modulation in the TOR pathway (20). PP2Cs are also ubiquitous and highly conserved in various pathogenic fungi, including the opportunistic human pathogen *Aspergillus fumigatus* and the plant pathogens *Botrytis cinerea*, *Fusarium graminearum,* and *Fusarium oxysporum* (18, 23 – 25). For example, PtcB, a PP2C phosphatase in *A. fumigatus*, is precipitated during MpkA dephosphorylation, implying that PtcB is key in the response to cell wall damage. The Δ*ptcB* mutant is avirulent at low doses in murine infection (25). In *B. cinerea*, the BcPTC1 and BcPTC3 mutants exhibit significantly attenuated virulence and inhibited mycelial growth (24). The targeted inactivation of PTC6 in *F. oxysporum* results in decreased virulence and enhanced susceptibility to cell wall stress (23). Additionally, ΔFgPTC1 weakens the virulence of *F. graminearum* in wheat (26). Thus, the most promising pharmacological targets following the hyper-activation/dysfunction of PP2Cs are linked to the virulence and growth of filamentous pathogenic fungi (25, 27 – 29). Fortunately, small molecule inhibitors of PP2C, such as the potent and specific inhibitor sanguinarine chloride, are targeted to the PP2C-type phosphatase domain for inactivation (29 – 31). The antifungal activity of sanguinarine affects the destruction of membrane integrity and the leakage of cellular content in the phytopathogenic fungi (32). Sanguinarine exhibited fungicidal activity against eight phytopathogenic fungi, including *Botrytis cinerea*, *Fusarium graminearum*, *F. oxysporum*, and *Magnaporthe oryzae* (33).

*Aspergillus flavus* is a saprophytic plant pathogenic fungus that contaminates various economically important crops, such as maize, peanuts, and tree nuts with carcinogenic aflatoxins (34). Phosphorylated proteins in *A. flavus* are involved in various biological processes (35). Serine phosphorylation (pS) is the most strongly represented (81.1%), followed by threonine phosphorylation (pT; 16.4%) with 598 phosphorylation sites (35). Studies on the various subtypes of *A. flavus* phosphatases*,* such as the dual-specificity C-terminal domain (CTD) phosphatase Ssu75 and tyrosine phosphatases Msg5 and Yvh1, have described the key roles of these phosphatases in MAPK signaling, fungal development, aflatoxin production, and pathogenicity (36 – 38). However, little is known about the functions of PP2C S/T phosphatase in *A. flavus*. Here, we aimed to identify PP2C phosphatases for PGK1 and demonstrate their roles and contributions in regulating development and virulence in *A. flavus*. Additionally, we revealed, for the first time, that Ptc1 and Ptc2 phosphatases participate in autophagy and mitochondrial pyruvate metabolism via PGK1 dephosphorylation in *A. flavus*, thus regulating aflatoxin synthesis. Our results may provide potential targets for controlling crop infections caused by fungal pathogens.

## RESULTS

### Identification of PP2C phosphatase homologs in *A. flavus*

**Fig 1:**
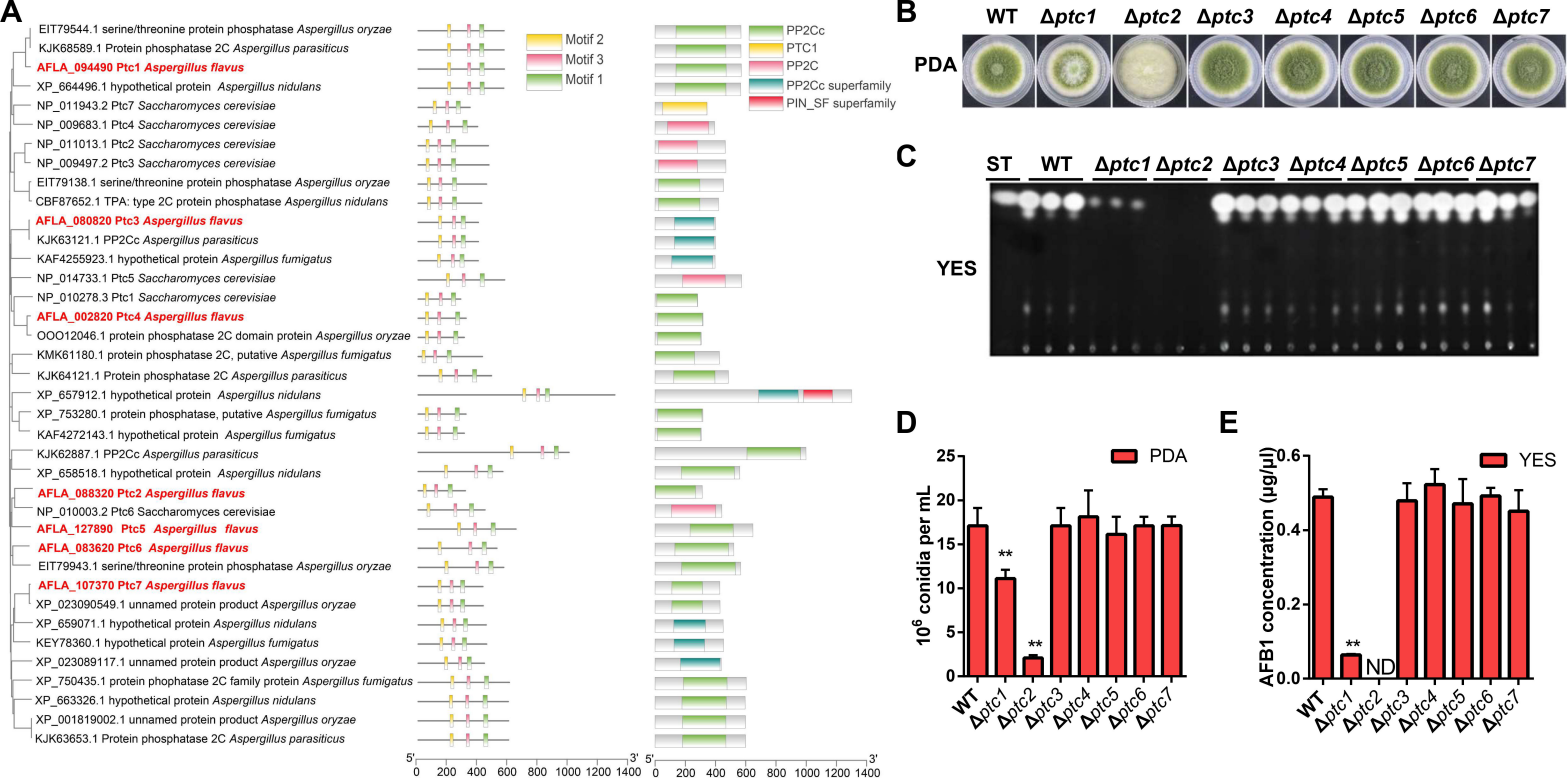
Identification of PP2Cs in *A. flavus*. (**A**) The phylogenetic relationships among PP2Cs and six species were analyzed. The PP2Cs of *A. flavus* are highlighted in red markers. Motifs and domains from PP2Cs were identified using the MEME/MAST GUI Wrapper and National Center for Biotechnology Information (NCBI) Batch CD search tool. The structural features and phylogenetic relationships were visualized using the “Gene Structure View” function in the software TBtools. (**B**) Colony morphology of wild-type (WT) and PP2C deletion strains cultured on potato dextrose agar (PDA) media at 37℃ for 5 days. (**C**) Thin-layer chromatography analysis of aflatoxin B1 (AFB_1_) in the WT and PP2C deletion strains. All strains were grown on yeast extract-sucrose (YES) liquid media at 29℃ for 5 days. (**D**) Conidial quantification in the WT and PP2C deletion strains as described in panel **B**. Mean ± standard deviation (SD) for three independent biological replicates is shown, and statistical significance was assessed by one-way analysis of variance (ANOVA) and Dunnett-T *post hoc* test. Asterisk represents significant difference (** indicates *P*  ≤  0.01). (**E**) AFB_1_ quantitative analysis of the WT and PP2C deletion strains as described in panel **C**. Data are the mean  ±  SD from three independent biological replicates (*n*  =  3); *P  ≤  0.05, **P  ≤  0.01, and ND represents not detected (analyzed by a one-way ANOVA with Dunnett’s test for multiple comparisons).

To identify PP2C phosphatases in *A. flavus*, we performed a homology-based BLAST (https://blast.ncbi.nlm.nih.gov/Blast.cgi) search using seven *S. cerevisiae* PP2C phosphatase protein sequences with deeply annotated PP2C phosphatases. Seven putative PP2C phosphatase proteins were identified and designated as Ptc1–Ptc7 in *A. flavus*: AFLA_094490 (*ptc1*), AFLA_088320 (*ptc2*), AFLA_080820 (*ptc3*), AFLA_002820 (*ptc4*), AFLA_127890 (*ptc5*), AFLA_083620 (*ptc6*), and AFLA_107370 (*ptc7*). A phylogenetic analysis and domain prediction of Ptc1–Ptc7 revealed that their phylogenetic relationship was better correlated with *Aspergillus* and contained a conserved 2C -type S/T phosphatase catalytic (PP2Cc) domain (Fig. 1A). These phosphatases have three conserved signature motifs (Motif 1 contains DG at position 14/15, Motif 2 has DG at position 10/11, and Motif 3 shows GD at position 10/11) that are involved in metal binding, coordination, and catalysis (Fig. 1A; Fig. S1A). These results indicate that the seven putative proteins are PP2C phosphatases, and the comparison results show that the phosphatase domains are conserved in fungi.

The *ptc1–ptc7* genes are located on chromosomes 1–5 of *A. flavus* (Fig. S1B). The expression profiles of the encoding genes were tested under various conditions induced using different media, including conidia-inducing media (potato dextrose agar[PDA]), aflatoxin-inducing media (yeast extract-sucrose[YES]), hyphal growth media (minimal medium [MM]), sclerotia-inducing media (yeast extract-peptone-dextrose, YPD) (Fig. S1C), and autophagy-inducing media MM with the TOR inhibitor rapamycin or the alkylating agent methyl methanesulfonate (MMS) (Fig. S1D). The expression patterns indicate that these PP2C phosphatase genes in *A. flavus* have different expression levels in hyphal growth, development, aflatoxin synthesis, and autophagy induction culture conditions.

To investigate the importance of PP2Cs in *A. flavus*, colony morphology assays were performed (Fig. S2A). The hyphal growth of the wild-type (WT) *A. flavus* cultured with 1 or 10 µM sanguinarine (a specific protein phosphatase 2C inhibitor) did not show marked changes compared with that of the untreated strain. However, the hyphal growth of the WT was severely blocked after the addition of 50 or 100 µM sanguinarine compared with that of the untreated WT (Fig. S2B). The conidiophore formation of the WT after sanguinarine stress was significantly reduced in a dose-dependent manner (Fig. S2C). Additionally, aflatoxins are important factors for evaluating the toxicity of *A. flavus* (39). Therefore, we examined aflatoxin production after treatment with different concentrations of sanguinarine (Fig. S2D). Thin-layer chromatography (TLC) assays indicate that PP2C inactivation after adding 50–100 μM sanguinarine failed to produce aflatoxin (Fig. S2D through E). We, therefore, speculated that there are essential phosphatases in regulating conidiophore formation and aflatoxin synthesis in *A. flavus*. To test this hypothesis, we generated the corresponding deletion mutants (designated as Δ*ptc1*, Δ*ptc2*, Δ*ptc3*, Δ*ptc4*, Δ*ptc5*, Δ*ptc6,* and Δ*ptc7*) (Fig. S3A through D). These mutants were screened using PCR and qPCR (Fig. S3C through D) and selected to clarify their functions based on the colony phenotype and aflatoxin production. As shown in Fig. 1B, Δ*ptc1* and Δ*ptc2* showed noticeable variations in the colony morphology compared to WT. The conidia number of the Δ*ptc1* and Δ*ptc2* mutants was decreased by 30% and 82%, respectively, compared to that of the WT (Fig. 1B and D). Similarly, aflatoxin production of Δ*ptc1* and Δ*ptc2* was conspicuously decreased compared to that of the WT strain (Fig. 1C and E), and *Ptc2* deletion resulted in undetectable aflatoxin production in *A. flavus*. As shown in Fig. S4, the result of the protein sequence alignments indicated that 52% homology between the Ptc1 and Ptc2 was presented in multiple conservative domain proteins with redundant functions. In summary, our data corroborated that these PP2Cs were highly conserved, and *ptc1* and *ptc2* as key phosphatases were essential to activate development and aflatoxin biosynthesis in *A. flavus*.

### Effects of *ptc1* and *ptc2* deletions on fungal growth, development, and aflatoxin synthesis

**Fig 2:**
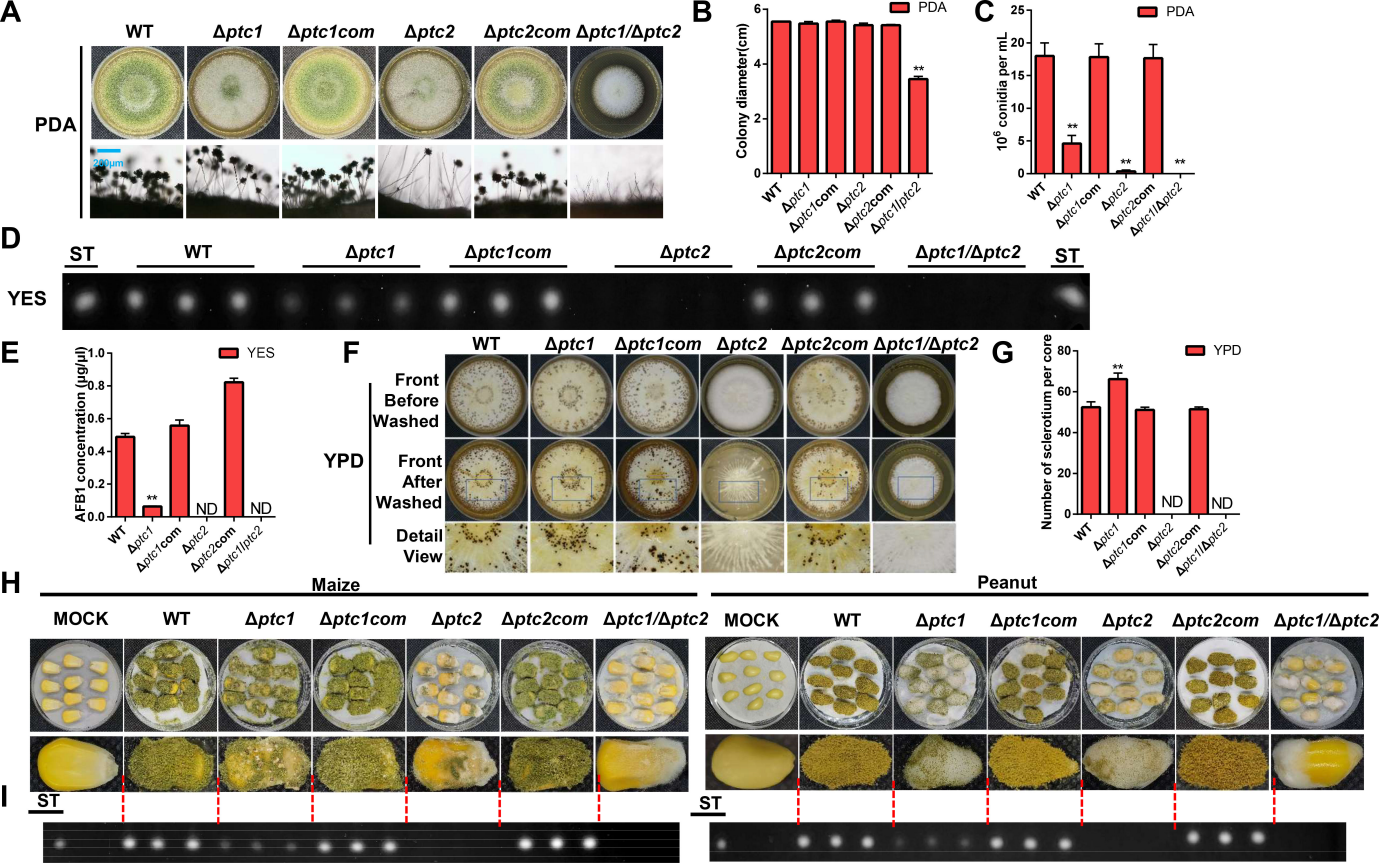
Phenotypic analysis of the Ptc1 and Ptc2 phosphatase mutants in *A. flavus*. (**A**) Point-inoculated cultures of *A. flavus* WT, Δ*ptc1*, Δ*ptc1*com, Δ*ptc2*, Δ*ptc2*com, and Δ*ptc1*/Δ*ptc2* strains on PDA media. These strains were cultivated at 37℃ for 5 days. The conidiophores of the WT and mutants were observed using differential interference contrast (DIC) microscopy. (**B**) Growth diameter of the WT, Δ*ptc1*, Δ*ptc1*com, Δ*ptc2*, Δ*ptc2*com, and Δ*ptc1/*Δ*ptc2* strains on PDA. Error bars represent ± SD from three independent experiments replicates. Statistical analyses were performed by a one-way ANOVA followed by Dunnett-T *post hoc* test. ** indicates *P* ≤  0.01. (**C**) The conidia were counted on PDA media after 5 days using microscopy. Error bars represent ± SD from three independent experiments replicates. One-way ANOVA and Dunnett *t*-test were used for statistical analyses. ** indicates *P* ≤  0.01. (**D**) TLC analysis of AFB_1_ production from the WT and mutants grown on YES liquid media after 6 days. (**E**) Relative quantification of AFB_1_ production in all the strains as mentioned in panel **D**. Error bars represent ± SD from three independent experiments replicates. ** indicates a significance level of *P* ≤  0.01 based on one-way ANOVA with Dunnett *t*-test and ND represents not detected. (**F**) The phenotypes of the WT and mutants were determined after they had grown on sclerotia-inducing medium (YPD) at 37℃ for 9 days. (**G**) The sclerotia were quantified in the YPD media after 9 days at 37℃. Error bars represent SD from three separate replicates. ** above the bars represent significantly different results and ND represents not detected. Above data were analyzed by a one-way ANOVA followed by Dunnett *t*-test. (**H**) Morphology of infected seeds in the WT and single- and double-knockout strains cultured at 29℃ for 5 days. (**I**) TLC results of aflatoxin production in the infected seeds.

Before the functional characterization of Ptc1 and Ptc2, their complementary strains (Δ*ptc1*com and Δ*ptc2*com) were used to prove that the function was fully re-obtained in their complementary strains Δ*ptc1*com and Δ*ptc2*com, respectively. To determine whether the functions of Ptc1 and Ptc2 are redundant, double-deletion mutants (Δ*ptc1*/*ptc2*) were constructed in *A. flavus* (Fig. S3A through D). The complemented *ptc1* in Δ*ptc1* and *ptc2* in Δ*ptc2* showed normal growth diameter, spore formation (Fig. 2A through C), and aflatoxin levels (Fig. 2D and E), similar to those in the WT. Notably, the colony growth diameter was significantly inhibited in the Δ*ptc1/ptc2* mutant compared to that in the single-knockout strains (Fig. 2A and B). All the deletion mutants (Δ*ptc1,* Δ*ptc2*, and Δ*ptc1/ptc2*) had a reduced number of conidia and conidiophores and decreased aflatoxin production compared to the WT and complemented strains (Fig. 2A, C, D and E). Δ*ptc1/ptc2* also displayed significant defects in these phenotypes compared to those in the single-knockout strains. The transcript levels of the conidia-related genes (*brlA* and *abaA*), aflatoxin biosynthesis structure genes (*aflO* and *aflP*), and globally regulated genes (*aflR* and *aflS*) were then measured using qRT-PCR. The expression levels of *brlA*, *abaA, aflO, aflP, aflR,* and *aflS* were significantly decreased in the Δ*ptc1*, Δ*ptc2*, and Δ*ptc1/ptc2* mutants compared to those in the WT and complemented strains (Fig. S5A and B).

Sclerotia resist damage in harsh environments and improve the survival rates of pathogenic fungi. We found that sclerotia formation was severely blocked in the Δ*ptc2* and Δ*ptc1*/*ptc2* mutants compared with that in the WT strains (Fig. 2F and G). However, the Δ*ptc1* and Δ*ptc2* mutants exhibited opposite regulatory effects on sclerotia production. Sclerotia formation was increased significantly in Δ*ptc1* compared to that in the WT. However, sclerotia was not formed in the absence of *ptc2*. The expression of the genes related to sclerotia regulation (*nsdD* and *sclR*) was further determined using qRT-PCR. The expression of *nsdD* and *sclR* in Δ*ptc1* showed a reversed trend compared to that in Δ*ptc2* and Δ*ptc1/ptc2*, consistent with the sclerotia production observed in Δ*ptc1*, Δ*ptc2*, and Δ*ptc1*/*ptc2* (Fig. S5C). The above results suggest that in *A. flavus*, *ptc2* is more involved and dominant in sclerotia formation than *ptc1*. Both Δ*ptc2* and Δ*ptc1/ptc2* showed analogous functions in the most severe phenotypes, almost abolishing conidia and sclerotia formation and aflatoxin synthesis.

### Effects of *ptc1* and *ptc2* deletions on pathogenicity to crop seeds

In *A. flavus*, pathogenicity to plant seeds mainly depends on conidial formation and aflatoxin production (40). Given that Δ*ptc1,* Δ*ptc2,* and Δ*ptc1*/*ptc2* exhibited serious defects in conidia and aflatoxin production, peanut and maize seeds were inoculated with spore suspensions from the WT, Δ*ptc1*, Δ*ptc2*, Δ*ptc1*/*ptc2,* and complemented strains. As shown in Fig. 2H; Fig. S6A, the pathogenicity of Δ*ptc1,* Δ*ptc2,* and Δ*ptc1/ptc2* on the peanuts and maize was significantly reduced compared to that of the WT and complemented strains. The number of spores on the infected peanuts and maize seeds was significantly reduced in the single- knockout strains, compared to that in the WT. Furthermore, the Δ*ptc1*/Δ*ptc2* strain displayed weaker conidiation than Δ*ptc1* and Δ*ptc2* in the peanuts and maize seeds (Fig. 2H; Fig. S6A). Aflatoxin was then extracted from the infected seeds, and TLC assays showed that Δ*ptc1*, Δ*ptc2,* and Δ*ptc1*/*ptc2* produced less aflatoxin in the peanuts and maize seeds than the WT and complemented strains did (Fig. 2I; Fig. S6B). These findings were consistent with the lower conidial and aflatoxin production in the PDA and YES liquid media *in vitro*. The above results demonstrated that both Ptc1 and Ptc2 functioned as positive regulators of conidial formation and aflatoxin biosynthesis in both the media and infected seeds, contributing to the pathogenicity of *A. flavus* to crop seeds.

### Ptc1 and Ptc2 are involved in autophagy

**Fig 3:**
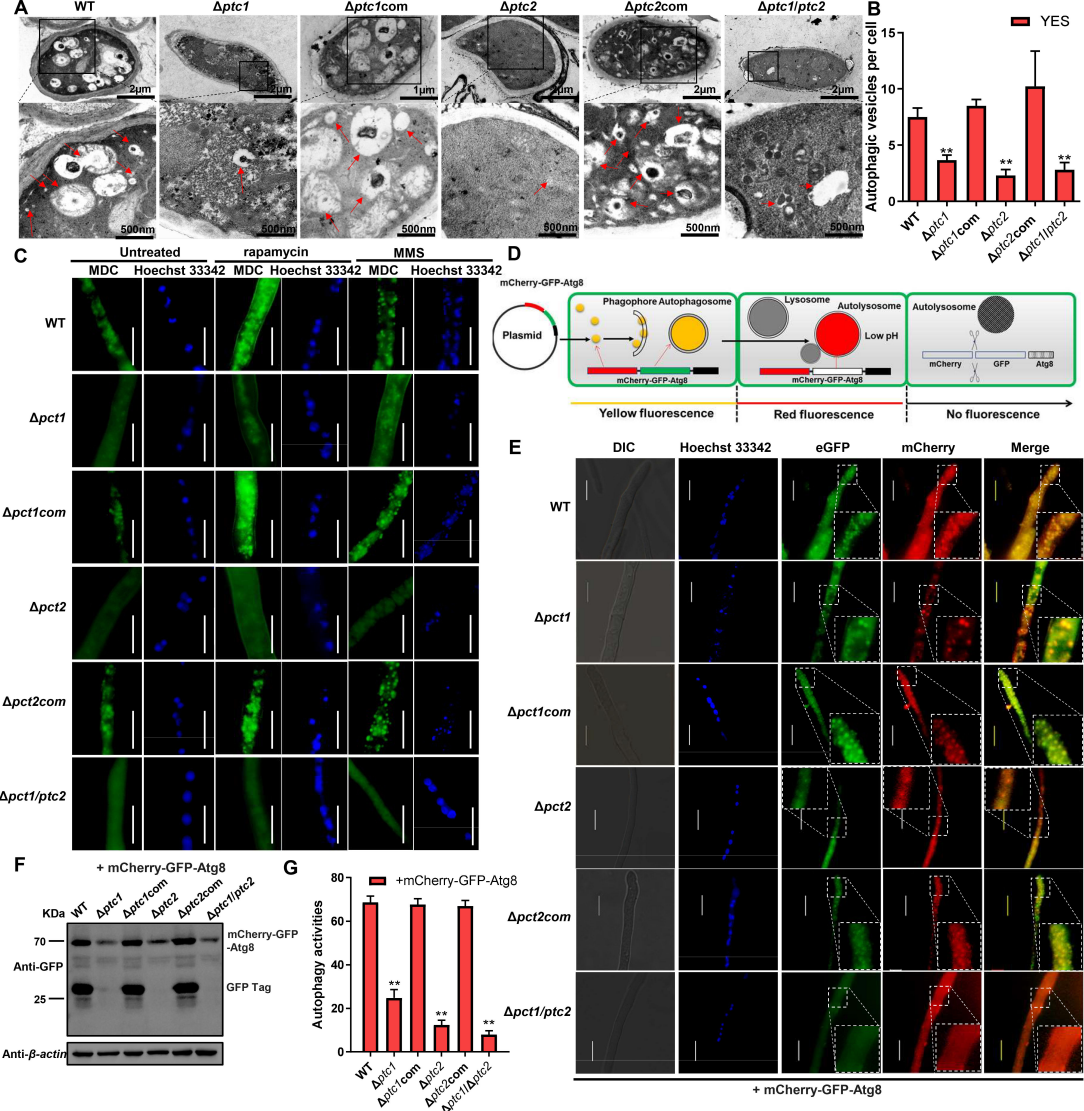
Ptc1 and Ptc2 phosphatases promote DNA damage- and rapamycin-induced autophagy in *A. flavus*. (**A**) Representative TEM images of autophagosomes in the WT, Δ*ptc1*, Δ*ptc1*com, Δ*ptc2*, Δ*ptc2*com, and Δ*ptc1*/Δ*ptc2* strains for 5 days. Red arrow, autophagic vesicles; scale bar, 500 nm. (**B**) The average number of autophagic vesicles per cell was measured. Error bars represent SD and ** represents *P* ≤  0.01. Above data were analyzed by a one-way ANOVA followed by Dunnett *t*-test. (**C**) Autophagic vesicles in the WT, Δ*ptc1*, Δ*ptc1*com, Δ*ptc2*, Δ*ptc2*com, and Δ*ptc1/Δptc2* strains were observed using MDC stains after rapamycin and MMS treatments. The nucleus of mycelium was stained with Hoechst 33342 (scale bar, 10 µm). (**D**) Tracking different stages of autophagy process with mCherry-GFP-Atg8. Schematic drawing showing the mCherry-GFP-Atg8 plasmid expressed in *A. flavus*, autophagosomes display both GFP (acid-sensitive tag) and mCherry (acid-insensitive tag) fluorescence (merge: yellow), whereas autolysosomes display only mCherry fluorescence (merge: red). Finally, during fusion of autophagosomes to late endosomes or lysosomes, red fluorescence was also quenching due to degradation of double-tagged protein. (**E**) Autophagosome of WT, Δ*ptc1*, Δ*ptc1*com, Δ*ptc2*, Δ*ptc2*com, and Δ*ptc1*/Δ*ptc2* expressing mCherry-eGFP-Atg8 strains was observed in *A. flavus*. Representative images of GFP and mCherry fluorescence appear in YES medium for 24  h from vegetative hypha. The nucleus was stained with Hoechst 33342. Scale bars, 10 µm. (**F**) Immunoblotting assays for WT, Δ*ptc1*, Δ*ptc1*com, Δ*ptc2*, Δ*ptc2*com, and Δ*ptc1/Δptc2* expressing mCherry-eGFP-Atg8 strains in YES medium for 48 h. The free GFP/total GFP ratios were quantified to determine autophagy activity, and β-actin was used as a loading control. (**G**) The comparison of autophagy activities quantified by immunoblotting assays of panel F. Each sample was standardized relative to its corresponding β-actin. Error bars represent SD. Above data were analyzed by a one-way ANOVA followed by Dunnett *t*-test (**P  ≤  0.01).

**Fig 4:**
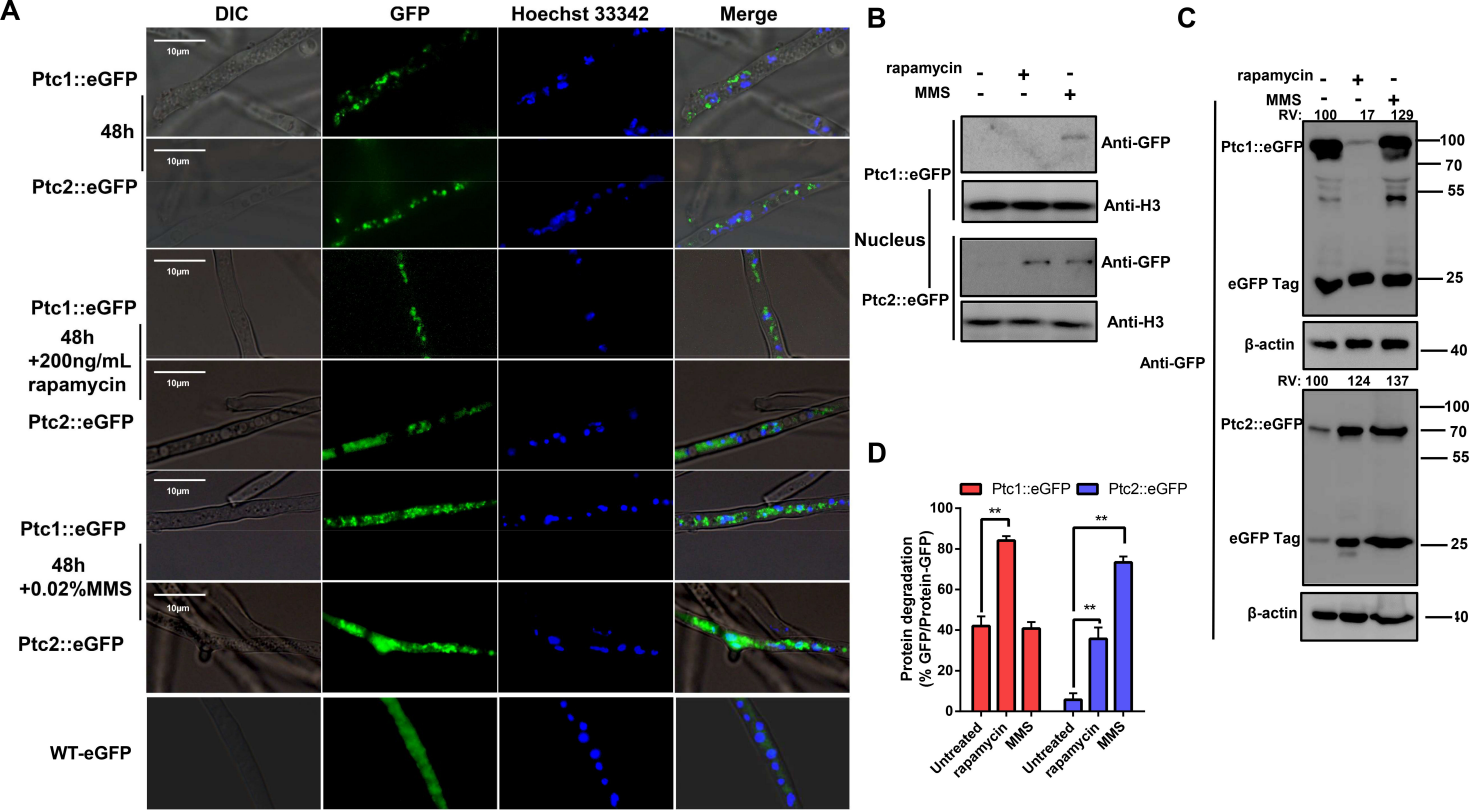
Degradation of Ptc1 and Ptc2 depends on core autophagy-induced processes in *A. flavus*. (**A**) Subcellular localization of Ptc1 and Ptc2. Micrographs of Hoechst 33342-stained hyphal cells expressing Ptc1:eGFP and Ptc2:eGFP in WT. Hyphal cells were collected from YES cultures after 48 h of treatment with 200 ng/mL rapamycin or 0.02% MMS. Note that fluorescent dye color and expressed fusion color are merged. Scale bar, 10 µm. (**B**) Ptc1 and Ptc2 proteins in the nucleus are detected by immunoblotting. The nucleus of Ptc1::eGFP and Ptc2::eGFP strains with 200 ng/mL rapamycin or 0.02% MMS for 4-h treatment and without treatment were extracted using a Nuclear Extraction Kit. Nuclear protein was separated by SDS-PAGE and detected by the anti-GFP antibody. Histone H3 as a loading control. (**C**) Ptc1::eGFP and Ptc2::eGFP strains were grown in YES media until they reached the early exponential phase and were then treated with 200 ng/mL rapamycin or 0.02% MMS for 4 h. Samples were blotted for GFP to quantify Ptc1 or Ptc2 fusion protein cleavage. Relative value (RV) shown in image, upper portion. The intensity of fusion protein in the untreated was defined as 100, and the RV of Ptc1 and Ptc2 levels of each sample was measured. The β-actin served as loading control for standardization of each relevant sample. (**D**) Band intensities of the fusion proteins Ptc1/Ptc2-GFP and free GFP were quantified using ImageJ software. GFP-Ptc1 and GFP-Ptc2 cleavage was calculated as the ratio of free GFP band to total GFP signal in the lane for three independent experiments. β-actin served as loading control for standardization of each relevant sample. Error bars represent SD and ANOVA (Dunnett *t*-test) was used for the comparison of means of multiple samples (*P ≤ 0.05 and **P ≤ 0.01).

The transcription levels of *ptc1-* and *ptc2-*encoding genes showed varying degrees of change in the autophagy-inducing media (Fig. S1D). Thus, we speculated that these two phosphatases may be involved in autophagy. To understand whether Ptc1 and Ptc2 regulate autophagy, we investigated autophagic vesicle formation and clearance *in vivo*. Compared with the WT and complemented strains, the Δ*ptc1,* Δ*ptc2,* and Δ*ptc1/ptc2* mutants had significant defects in autophagic vesicle formation (Fig. 3A and B) and a significantly reduced fluorescence intensity in monodansylcadaverine (MDC)-labeled autophagic vacuoles, which confirmed the autophagic defects (Fig. 3C; Fig. S7A and S7B). Autophagic vesicles in these mutants had observable defects even upon autophagy induction (Fig. 3C; Fig. S7A and S7B). Atg8, used as a marker for autophagy, is tightly associated with autophagosomes (41). Therefore, we successfully constructed a double-tagged Atg8 (mCherry-GFP-Atg8) expression system in the WT, Δ*ptc1*, Δ*ptc2*, Δ*ptc1*/*ptc2,* and complemented strains (Fig. S7C), and performed fluorescence microscopy and autophagy activity experiments using double-tagged Atg8 (Fig. 3D through G; Fig. S7D). The assay results showed that autophagosome and autolysosome formation in the Δ*ptc1*, Δ*ptc2,* and Δ*ptc1*/*ptc2* mutants were blocked compared to the WT and complemented strains. Autophagosome formation-related proteins were further detected, and immunoblotting confirmed that the levels of Atg5 and Atg12–Atg5 conjugate proteins were significantly decreased in the Δ*ptc1/ptc2* double mutant compared to those in the WT and complemented strains (Fig. S7E). These results suggest that both Ptc1 and Ptc2 promote autophagy in *A. flavus* (Fig. 3). Autophagy is pivotal for the degradation of autophagy-associated proteins and cell organelles. To investigate whether Ptc1 and Ptc2 were degraded following the induction of autophagy, we constructed green fluorescent protein (GFP) fusion protein strains (Ptc1::eGFP and Ptc2::eGFP), in which, Ptc1 or Ptc2 was fused to the GFP under its endogenous promoter (Fig. S8A). The WT-GFP strain was constructed as a control strain to express GFP alone under the control of the *ptc3* promoter in *A. favus*. (Fig. 4A; Fig. S8B and S8C). We verified the fusion of these proteins using subcellular localization experiments (Fig. 4A), diagnostic PCR (Fig. S8D), and western blotting (Fig. S8E). Both Ptc1 and Ptc2 were localized in the cytoplasm (Fig. 4A). An immunoblotting analysis revealed that the protein level of Ptc2 was significantly higher than that of Ptc1 in *A. flavus* (Fig. S8F) and that both Ptc1 and Ptc2 were degraded to different degrees, as indicated by the accumulation of free GFP (Fig. S8E).

We further treated Ptc1::eGFP and Ptc2::eGFP with rapamycin to induce macroautophagy or with MMS to induce DNA damage. Ptc1::eGFP and Ptc2::eGFP showed diffuse-type fluorescent particles in response to autophagy induced by rapamycin or MMS, compared to that under untreated conditions (Fig. 4A). Ptc1 and Ptc2 exhibited nuclear localization after MMS treatment, and Ptc2 also displayed evident nuclear penetration after the rapamycin treatment compared with no treatment (Fig. 4B). Ptc2 levels were significantly increased after the rapamycin and the MMS treatments (Fig. 4C). The Ptc1 levels were also significantly upregulated after the MMS treatment but were downregulated considerably after the rapamycin treatment. Furthermore, Ptc2 was significantly degraded after the rapamycin and MMS treatments compared to the untreated controls (Fig. 4C and D). However, the excessive degradation of Ptc1 was only induced by rapamycin, suggesting that the Ptc1 and Ptc2 levels may be strictly controlled by inducing autophagic degradation through rapamycin, while Ptc2 also participates in DNA damage-induced autophagic degradation.

### Metal ion-coordinating aspartate (Asp) residues of Ptc1 and Ptc2 are essential for catalytic activity

**Fig 5:**
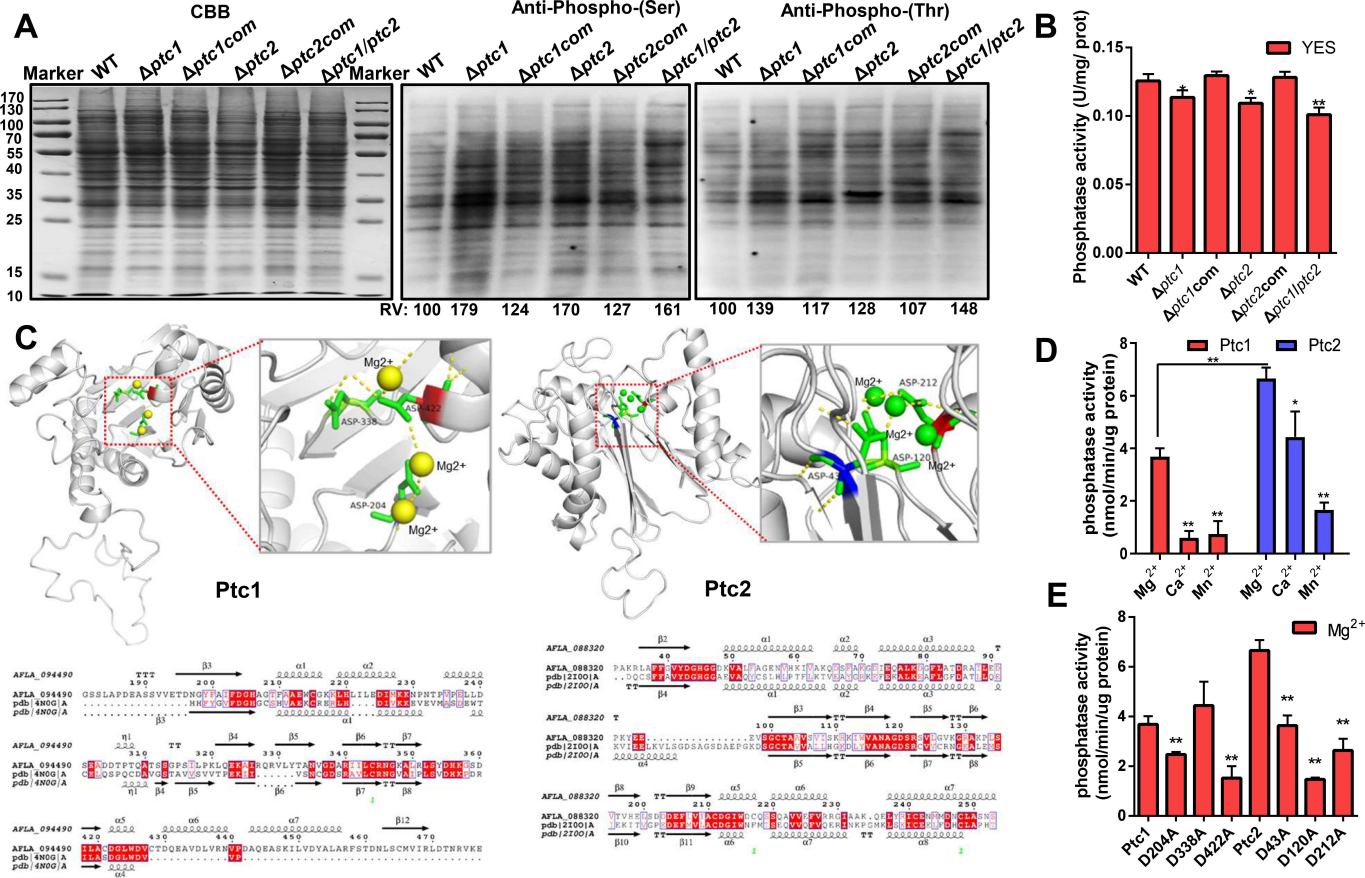
Catalytic activities of Ptc1 and Ptc2 *in vivo* and *in vitro*. (**A**) Profiling of Ser/Thr phosphorylation in the WT and all mutants. Total proteins (20 µg) were collected and detected by immunoblotting after SDS-PAGE using the anti-phospho-Ser and anti-phospho-Thr antibodies. Coomassie Brilliant Blue-stained gel served as loading control for standardization of each relevant lane. CBB is defined as Coomassie brilliant blue. The WT was defined as 100. The lane intensities were measured with Chemi Analysis software. (**B**) Phosphatase activity in WT, Δ*ptc1*, Δ*ptc1*com, Δ*ptc2*, Δ*ptc2*com, and Δ*ptc1*/Δ*ptc2* strains was detected using an alkaline phosphatase activity test kit. Mycelia from all the strains were homogenized and collected for enzymatic activity measurements *in vivo*. Error bars represent SD from three independent experiments. Statistical significance was determined by ANOVA (Dunnett *t*-test). * and ** represent *P* ≤ 0.05 and *P* ≤ 0.01, respectively. (**C**) Prediction of Ptc1 and Ptc2 enzyme active sites by SWISS-MODEL. Multiple sequences were aligned using CLC seq-view6 software. The secondary structural information of proteins was predicted using ESPRIT 3.0. (**D**) Analysis of the phosphatase activity of Ptc1 and Ptc2 using p-nitrophenyl phosphate-based phosphatase assays. Ptc1- or Ptc2-His fusion proteins were expressed and purified in *E. coli*. The absorbance of dephosphorylated p-nitrophenyl phosphate at 405 nm was detected after a reaction at 37℃ for 30 min. Error bars represent SD from three independent experiments. * and ** represent *P* ≤ 0.05 and *P* ≤ 0.01, respectively. The data were analyzed by two-way ANOVA followed by Tukey’s *post hoc* test. (**E**) Analysis of the phosphatase activity of Ptc1 and Ptc2 and their mutated versions in Mg^2+^ metal ion solutions. Error bars represent SD from three independent experiments. Above data were analyzed by a one-way ANOVA followed by Dunnett *t*-test. ** represent *P* ≤ 0.01.

PP2Cs play key roles in dephosphorylating S/T proteins (42). To assess the overall level of phosphorylation in the knockout strains (Δ*ptc1*, Δ*ptc2,* and Δ*ptc1*/*ptc2*), immunoblotting was performed (Fig. 5A). In comparison to the WT and complemented strains, the phosphorylated S/T levels of a few bands in Δ*ptc1*, Δ*ptc2*, and Δ*ptc1/ptc2* strains were increased, although the amount of total protein was almost the same. Then, the mycelia of the mutants were collected to determine phosphatase activity. The enzymatic activity *in vivo* showed that disrupting *ptc1* or *ptc2* caused slight decreases in phosphatase activity (Fig. 5B). Additionally, the phosphatase activity was significantly decreased in *Δptc1*/*ptc2*, suggesting that their functions overlap *in vivo* (Fig. 5B). PP2Cs are metal ion-dependent phosphatases that are coordinated by a universally conserved core of Asp residues (43). The protein structures and metal-coordinating residues of Ptc1 and Ptc2 were constructed and calculated using the alignment PDB|4N0G|A and PDB|210O|A, respectively (Fig. 5C). The structures that displayed metal ions in the active site were coordinated by residues aligning with Ptc1 Asp204, Asp338, and Asp422, and those aligning with Ptc2 Asp43, Asp120, and Asp212. To test these candidates, the residues Asp204, Asp338, and Asp422 of Ptc1 and Asp43, Asp120, and Asp212 of Ptc2 were mutated to alanine (Ala) and expressed in *Escherichia coli* cells (Fig. S9A and B). To estimate the structural basis of the thermal stability between the WT and the metal ion-coordinating mutants of Ptc1 and Ptc2, we measured the melting temperature (Tm) with the Nano differential scanning calorimetry (Nano-DSC) instrument (Calorimetry Sciences Corp. Lindon, UT, USA). This result indicates that the thermal stability of Ptc1 Asp204, Asp338, and Asp422 to A is higher than that of the WT of Ptc1 (Fig. S9C). For Ptc2, only the thermal stability of Ptc2 Asp43 to A is lower compared to the WT. This result shows that mutation Asp into A affects the structural stability of phosphatase, changing the enzyme activity. *In vitro*, Ptc1 and Ptc2 showed high phosphatase activity in the presence of Mg^2+^ (Fig. 5D). The steady-state phosphatase activity of the His-tag WT and site mutant proteins was tested in a solution containing Mg^2+^ (Fig. 5E). Ptc1 Asp204 and Asp422 and Ptc2 Asp43, Asp120, and Asp212, in which the highly conserved metal-binding site (Asp) was mutated to an inactive form (Ala), had lost their phosphatase activity (Fig. 5E). Ptc1 and Ptc2 are therefore Mg^2+^-dependent protein phosphatases. The metal ion-coordinating residues of Ptc1 (D204 and D422) and Ptc2 (D43, D120, and D212) are important for catalytic activity *in vitro*.

### Metal ion-coordinating Asp residues of Ptc1 and Ptc2 are essential for development and aflatoxin synthesis in *A. flavus*

**Fig 6:**
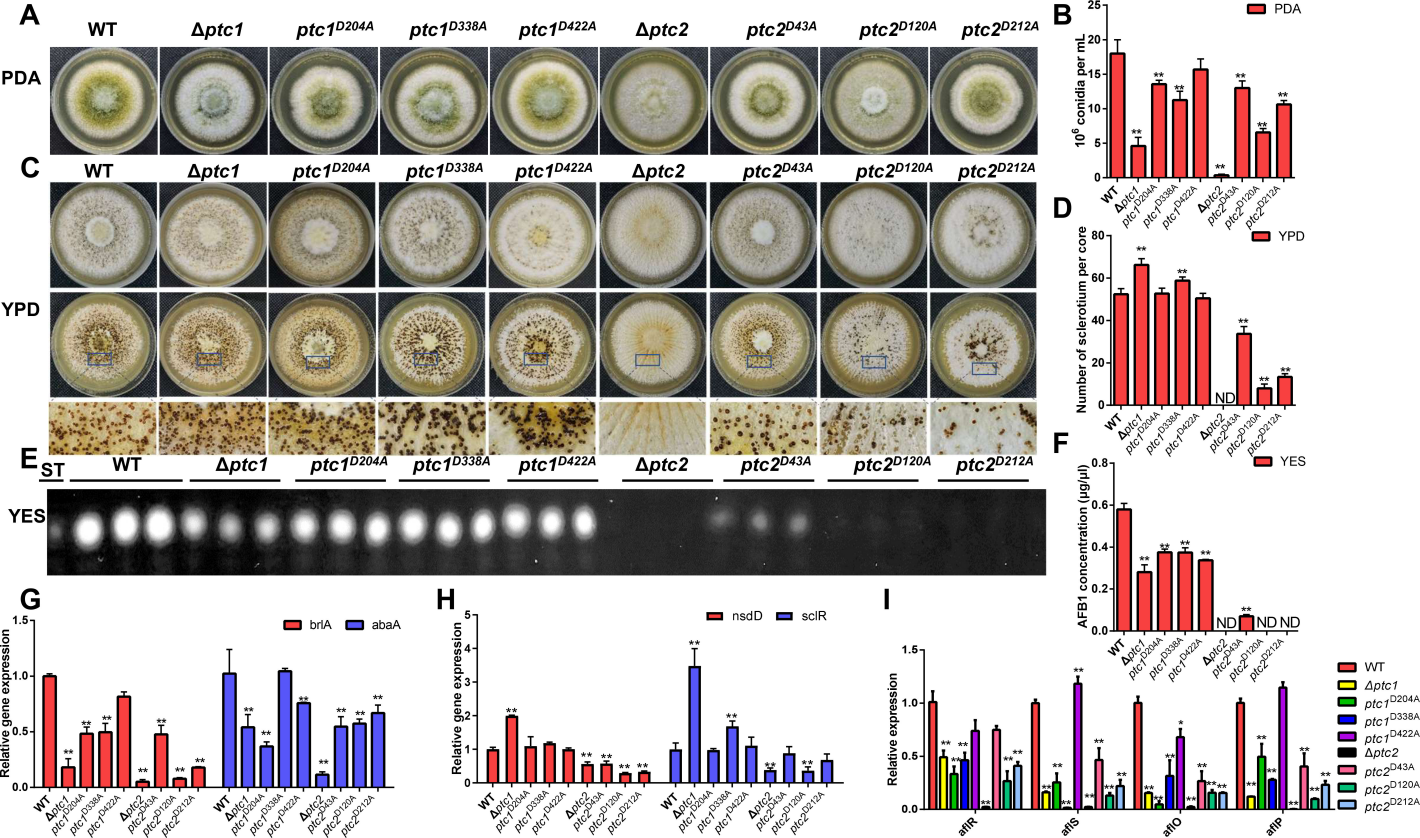
Phenotypic analysis of Ptc1 and Ptc2 phosphatase point mutations in *A. flavus*. (**A**) Point-inoculated cultures of *A. flavus* WT, Δ*ptc1*, *ptc1^D204A^*, *ptc1^D338A^*, *ptc1^D422A^*, Δ*ptc2*, *ptc2^D43A^*, *ptc2^D120A^*, and *ptc2^D212A^* strains on PDA at 37℃ for 5 days. (**B**) The conidia numbers of WT and all mutants were counted by microscopy. Error bars represent SD from three independent experiments. Above data were analyzed by a one-way ANOVA followed by Dunnett *t*-test. ** represent *P* ≤ 0.01. (**C**) The phenotypes of strains were determined after growth on sclerotia-inducing media (YPD) at 37°C for 9 days (**D**) The number of sclerotia was measured from YPD as mentioned in (**C**). Error bars represent SD from three independent experiments. Above data were analyzed by a one-way ANOVA followed by Dunnett *t*-test. ** represent *P* ≤ 0.01. (**E**) TLC analysis of AFB_1_ production from the WT and mutants on YES liquid media after 6 days. (**F**) Relative quantification of AFB_1_ production in the strains from YES liquid media. Error bars represent SD from three independent experiments. Above data were analyzed by a one-way ANOVA followed by Dunnett *t*-test. ** represent *P* ≤ 0.01. (**G**) Transcriptional levels of conidia-related genes *brlA* and *abaA*. (**H**) Transcriptional levels of the sclerotia-specific genes *nsdD* and *sclR*. (**I**) Relative expression levels of aflatoxin biosynthesis regulatory and structural genes *aflR*, *aflS*, *aflO*, and *aflP* determined by qRT-PCR after 48 h. Above data were analyzed by a one-way ANOVA followed by Dunnett *t*-test. *P ≤ 0.05, **P ≤ 0.01. Experiments were repeated three times. Error bars represent SD.

To further investigate the function of the catalytic sites of Ptc1 and Ptc2 *in vivo*, active site mutants were constructed, and the site-specific mutations were verified using PCR (Fig. S3C) and gDNA sequencing (Fig. S9D). Compared with the Δ*ptc1* and Δ*ptc2,* the resulting Ptc1 ^D338A^ and all the Ptc2 site mutants showed similar results in terms of conidiation (Fig. 6A and B), sclerotia formation (Fig. 6C and D), and aflatoxin biosynthesis (Fig. 6E and F), implying that the D338 of Ptc1 and the D43, D120, and D212 of Ptc2 are important for their biofunctions. However, the site-specific mutation D422A in Ptc1 only impaired aflatoxin biosynthesis. Compared with the WT, the D204A of Ptc1 was significantly inhibited in sporulation (Fig. 6A and B) and aflatoxin production (Fig. 6E and F).

We further examined the transcript levels of genes related to conidia production (*brlA* and *abaA*), sclerotia formation (*nsdD* and *sclR*), and aflatoxin biosynthesis (*aflO* and *aflP*) and the globally regulated genes (*aflR* and *aflS*) in all the mutants. *brlA* and *abaA* were significantly downregulated in the site-specific mutations of Ptc1 (D204A) and Ptc2 (D43A, D120A, and D212A) compared to those in the WT (Fig. 6G). However, D338A only downregulated the conidia-related gene *brlA* compared to that in the WT (Fig. 6G). These results indicate that the D204A and D338A of Ptc1 and all the site-specific mutations of Ptc2 display their gene deletion mutant-like phenotypes during conidiospore formation, suggesting that these Asp residues maintain the integral functions of Ptc1 and Ptc2 in *A. flavus*. Among the sclerotia-related genes, *nsdD* was severely suppressed in all the site-specific mutations of Ptc2, consistent with the Ptc2 deletion (Fig. 6H). Only the *sclR* of the D338A mutation was prominently upregulated compared with that of the other site-specific mutations of Ptc1 (Fig. 6H). These results suggest that D338 of Ptc1 and all three Asp residues of Ptc2 play critical roles in sclerotia formation. The qPCR data showed that the transcript levels of AFB_1_ cluster genes (*aflS, aflO*, and *aflP*) were downregulated in all the site-specific mutations of Ptc2 (Fig. 6I). Similarly, *aflO* was downregulated in all the site-specific mutations of Ptc1 (Fig. 6I). All the site-specific mutants of Ptc1 and Ptc2 exhibited gene deletion mutant-like phenotypes in aflatoxin biosynthesis. These data demonstrate that the metal ion-coordinating Asp residues of Ptc1 and Ptc2 are important for development and aflatoxin synthesis in *A. flavus.*

### Identification of potential Ptc1- and Ptc2-binding proteins by immunoprecipitation-mass spectrometry (IP-MS)

**Fig 7:**
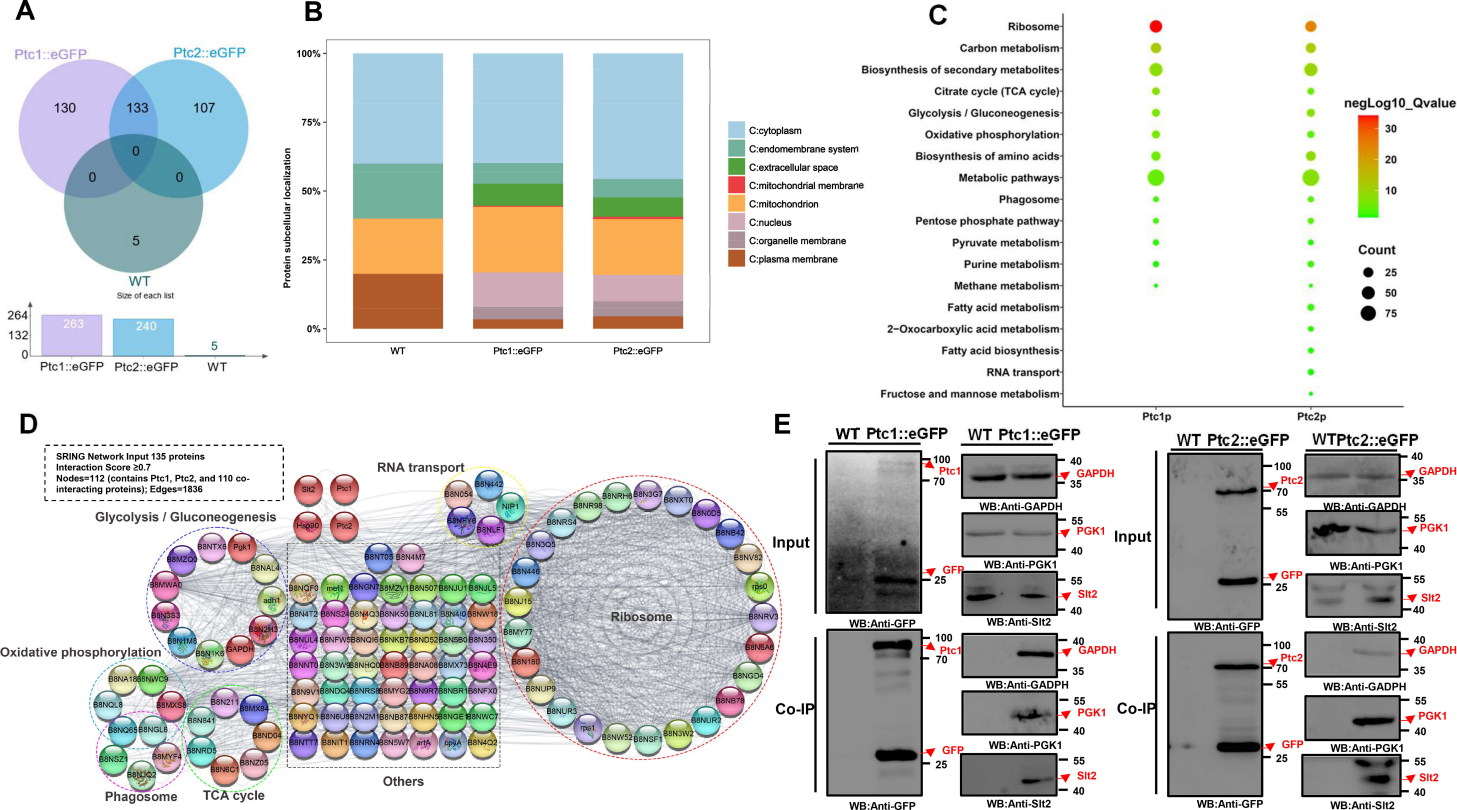
IP-MS revealed that phosphatases Ptc1p and Ptc1p regulate various biological processes in *A. flavus*. (**A**) Venn diagram showing the identified WT and Ptc1- and Ptc2-interacting protein numbers from the IP-MS data. *A. flavus* WT as negative control group. (**B**) Subcellular localization prediction of proteins identified by IP-MS in WT, Ptc1::eGFP, and Ptc2::eGFP groups. All the identified proteins were annotated using a web server for predicting protein subcellular localization BUSCA. (**C**) The bubble diagram shows the enriched KEGG pathways of all identified proteins from Ptc1::eGFP and Ptc2::eGFP groups, and setting the cut-off *Q-*value as 0.05 for significant enrichment with Benjamini-Hochberg correction. (**D**) Protein-protein interaction (PPI) networks of overlap interacting proteins of both Ptc1 and Ptc2. The interaction network contained 112 nodes (contains Ptc1, Ptc2, and 110 co-interacting proteins) under a high-quality interaction score ≥0.7 and hide disconnected nodes. The PPI network was generated from the STRING database v11.5 and visualized in Cytoscape. The six highly connected interaction clusters. The clusters were generated by stringApp tool in Cytoscape. (**E**) The proteins with high interaction scores, Slt2, PGK1, and glyceraldehyde-3-phosphate dihydrogen (GAPDH), were verified by co-immunoprecipitation experiments. Anti-GFP magnetic beads immunoprecipitated the proteins, which were further analyzed by immunoblotting with anti-GFP, GAPDH, PGK1, and Slt2 antibodies. Input sample was used as control.

To understand the regulatory mechanisms of Ptc1 and Ptc2, we examined the potential interacting proteins in stable Ptc1::eGFP and Ptc2::eGFP cells (Fig. 7; Fig. S10A; Table S3). The multiple proteins and GFP bands that co-immunoprecipitated with the full-length Ptc1::eGFP and Ptc2::eGFP, but not with the WT, were identified. The identification of the interacting proteins is shown in Table S4. Ptc1 and Ptc2 were more abundant in the IP-MS data of the Ptc1::eGFP and Ptc2::eGFP strains, respectively (Table S5). Neither appeared on the other list, suggesting that Ptc1 may not robustly interact with Ptc2 (Table S5). A list of 263 unique proteins interacting with Ptc1 and 240 interacting with Ptc2 was obtained (Table S4). Five false-positive proteins of the same type as the control WT were removed (Fig. 7A). We identified 133 proteins that co-interact with Ptc1 and Ptc2 (Fig. 7A). To understand the biological functions associated with these proteins better, the prediction of protein subcellular localization was performed by Bologna Unified Subcellular Component Annotator (BUSCA) web server (Table S6 and Fig. 7B). Most of the proteins were found to be important for biosynthesis and metabolism in the cytoplasm and mitochondria (Fig. 7B). Gene Ontology (GO) enrichment analysis showed that many of the identified Ptc1- and Ptc2-interacting proteins are enriched in biosynthetic process, cellular metabolic process, response to chemical, cell death, and protein-containing complex localization processes (*P*  ≤  0.05) (Fig. S10B and C; Table S7). Kyoto Encyclopedia for Genes and Genomes (KEGG) pathway enrichment analysis showed that these proteins are closely related to glycolysis/gluconeogenesis, carbon metabolism, and phagosomes (Fig. 7C; Table S8). Compartments enrichment analysis showed that these proteins are closely related to the cytoplasm,cytosolic ribosome, mitochondrion, mitochondrial protein complex, and proton-transporting two-sector ATPase complex, suggesting a conserved role of Ptc1 and Ptc2 in the regulation of mitochondrial and cytoplasmic-related proteins (Fig. S10D; Table S8). However, compared to the Ptc1-interacting proteins, the Ptc2-interacting proteins were specifically enriched in 2-oxocarboxylic acid metabolism, fatty acid biosynthesis, RNA transport, and fructose and mannose metabolism (Fig. 7C), indicating that Ptc2 may be important in regulating metabolic processes.

In total, 263 Ptc1 and 240 Ptc2 interactions were mapped to the protein interaction database and connected by 185/182 nodes and 4,104/3,306 edges, respectively (Fig. S11A, S11B; Table S9). The ribosome, phagosome, and glycolysis/gluconeogenesis pathways were enriched (Fig. S11A and S11B). Meanwhile, the 133 overlapping pivotal substrate proteins of Ptc1 and Ptc2 may perform overlapping biological function (Fig. 7D). Protein-protein interaction (PPI) screening and filtering identified 110 proteins as potential interacting molecules between Ptc1 and Ptc2, such as the centrally strong interacting protein mitogen-activated protein kinase (Slt2), glyceraldehyde-3-phosphate dihydrogen (GAPDH), and phosphoglycerate kinase PGK1 (interaction score ≥0.7), which constitute a complex and strong PPI network (Fig. 7D and E). Endogenous Slt2, GAPDH, and PGK1 were reciprocally and mutually immunoprecipitated and identified by western blotting (Fig. 7E). These results are consistent with those of the PPI network analysis, confirming the reliability of our IP-MS data. Importantly, the core PGK1 protein was identified as a high abundance protein in IP-MS (Fig. S12A through D; Table S5). These results indicate that the interaction proteins of Ptc1 and Ptc2 are involved in multiple biological processes and perform critical biological functions in *A. flavus.*

### Slt2 and PGK1 are dephosphorylated by Ptc1 and Ptc2 in *A. flavus*

**Fig 8:**
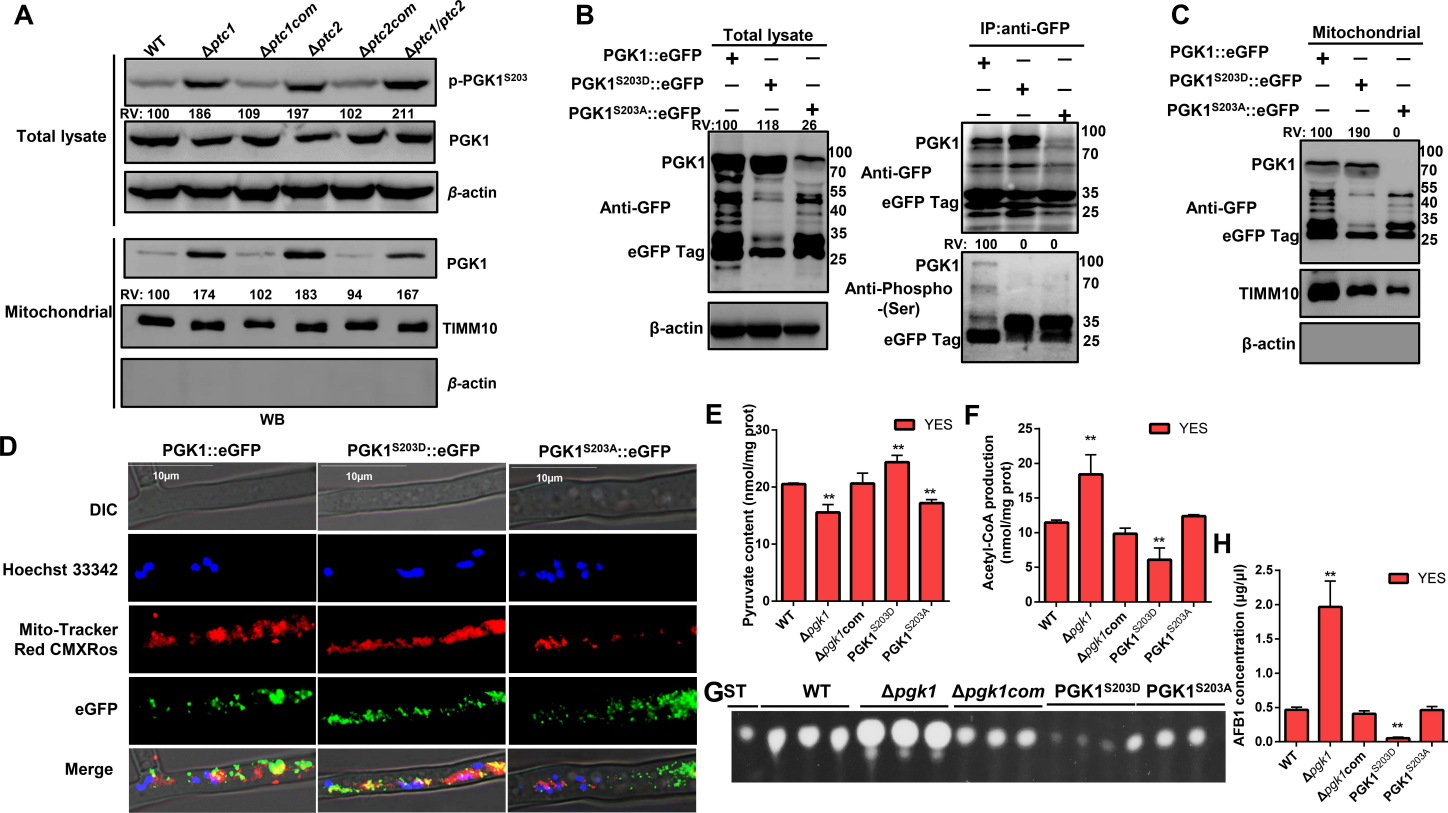
Ptc1- and Ptc2-mediated PGK1 dephosphorylation and mitochondrial translocation promote aflatoxin biosynthesis in *A. flavus.* (**A**) Isolated mitochondria from WT, Δ*ptc1*, Δ*ptc1*com, Δ*ptc2*, Δ*ptc2*com, and Δ*ptc1*/Δ*ptc2* strains followed by immunoblotting analyses with the anti-PGK1 and anti-phospho-PGK1S203. The relative expression levels of phospho-PGK1 and PGK1 were normalized by β-actin and TIMM10 of each group in total lysate and mitochondria, respectively. Then, the expression level of WT group was defined as 100. The β-actin is used to evaluate mitochondrial purity. (**B**) Phosphorylation of PGK1 *in vivo*. Total proteins were extracted from PGK1::GFP and PGK1 S203 site mutants at 29°C. The β-actin served as loading control. RV shown in image, upper portion. The intensity of fusion protein in PGK1 of WT was defined as 100, and RV of PGK1 level of each sample was measured. PGK1 S203A and S203D were immunoprecipitated with GFP magnetic beads, and phosphorylation of PGK1 was detected by immunoblotting. Anti-GFP immunoblotting was used to show equal loading. (**C**) Isolated mitochondria from PGK1::GFP and PGK1 S203 site mutants in *A. flavus* , followed by immunoblotting analyses with the indicated antibodies. RV shown in image upper portion. The intensity of fusion protein in PGK1 of WT was defined as 100, and RV of PGK1 level of each sample was measured. TIMM10 served as loading control in mitochondria. The β-actin is used to evaluate mitochondrial purity. (**D**) Subcellular localization of PGK1::GFP and S203 site mutant (S203D and S203A) fusion proteins. The localization of the fusion proteins was monitored after 48 h in YES liquid media. Hoechst 33342 and MitoTracker Red CMXRos were used to detect the nuclei and mitochondria, respectively. Scale bar, 10 µm. (**E**) All *pgk1* mutants were cultured in YES liquid media at 29℃ for 3 days. Levels of cytosolic pyruvate were measured. Error bars represent SD from three independent biological replicates. Above data were analyzed by a one-way ANOVA followed by Dunnett *t*-test. Asterisks represent significant differences (**P ≤ 0.01). (**F**) Mitochondrial fractions of the mutants were prepared, and the levels of mitochondrial glyceraldehyde-3-phosphate dihydrogen (acetyl-CoA) were measured. Mean ± standard deviation for three independent experiments. Above data were analyzed by a one-way ANOVA followed by Dunnett *t*-test. ** represent *P* ≤ 0.01. (**G**) TLC analysis of AFB_1_ production in PGK1 mutants after 6 days of incubation. (**H**) Quantitative analysis of AFB_1_, as shown in Fig. 8G. All experiments had three biological replicates. Error bars represent SD from three independent biological replicates. Above data were analyzed by a one-way ANOVA followed by Dunnett *t*-test. ** above the bars represents significantly different results (**P ≤ 0.01).

Located at the core of the above interaction network, Slt2 and PGK1 were shown to interact with Ptc1 and Ptc2 strongly. We speculated that Ptc1 and Ptc2 may promote redundant biological functions by mediating Slt2 and PGK1 dephosphorylation. Hence, we analyzed the phosphorylation status of endogenous Slt2 (Fig. S13A). Higher phosphorylation levels were observed in Δ*ptc1*, Δ*ptc2,* and Δ*ptc1/ptc2* than in the WT and complemented strains of *A. flavus*. Previous studies in our laboratory revealed that Slt2 dephosphorylation is involved in growth, development, and aflatoxin synthesis in *A. flavus* (44, 45). However, as another key node protein in the Ptc1 and Ptc2 interaction network and metabolic enzyme in the glycolysis/gluconeogenesis pathways in *A. flavus,* PGK1 and its functions remain poorly understood. We observed that PGK1^S203^ was hyperphosphorylated in Δ*ptc1*, Δ*ptc2,* and Δ*ptc1/ptc2* compared with that in the WT and complemented strains of *A. flavus* (Fig. 8A). Dephosphorylation assays *in vitro* revealed that Ptc1 and Ptc2 reduced the phosphorylation levels of PGK1 more than the inactivated and no-phosphatase treatments did, indicating that Ptc1 and Ptc2 may directly dephosphorylate PGK1 (Fig. S14A and B). The level of PGK1 phosphorylation sharply decreased when S203 was mutated to Ala *in vitro* compared to that of the WT PGK1 (Fig. S14C and E)*,* suggesting that the phosphorylation of S203 is the major phosphorylation site for PGK1. These findings suggest that Ptc1 and Ptc2 are involved in the dephosphorylation of Slt2 and PGK1 in *A. flavus*.

### Ptc1 and Ptc2 affect mitochondrial translocation of PGK1 through dephosphorylation of PGK1 S203

Phosphorylated PGK1 has dual roles in regulating glycolysis and mitochondrial metabolism (6). In this study, the deletion of Ptc1 and Ptc2 led to significantly increased S203 phosphorylation and the mitochondrial translocation of PGK1 in Δ*ptc1*, Δ*ptc2*, and Δ*ptc1/ptc2* compared to those in the WT and complemented strains (Fig. 8A). To determine whether the PGK1 S203 site has mitochondrial compartment-dependent functions, the conserved PGK1 phosphorylation site S203 was mutated to Ala (A) and aspartic acid (D) to mimic non-phosphorylated and constitutively phosphorylated states, respectively. These mutations were verified using PCR and DNA sequencing (Fig. S15A and B). The S203 site mutants showed extremely low Ser phosphorylation levels compared to those in the WT, validating that the modified S203 is the major site of phosphorylation in PGK1 *in vivo* (Fig. 8B). We further examined the subcellular distribution in the stable site-directed mutants (S203A and S203D) of PGK1. The PGK1^S203A^ did not enter the mitochondria, in contrast to WT PGK1 and PGK1^S203D^ (Fig. 8C). The subcellular localization analysis of PGK1 also confirmed that the GFP signal of PGK1 was localized to the mitochondria and cytoplasm (Fig. 8D). The GFP signals of PGK1^S203A^ did not indicate co-localization with the mitochondria. In contrast, those of PGK1^S203D^ were only localized in the mitochondria (Fig. 8D). Thus, the Ptc1- and Ptc2-mediated S203 dephosphorylation of PGK1 blocks the subsequent mitochondrial translocation of PGK1 in *A. flavus.*

### Effects of PGK1 dephosphorylation on aflatoxin biosynthesis

Mitochondrial pyruvate metabolism is regulated by two key enzymes, pyruvate dehydrogenase kinase and PGK1, which inactivate the pyruvate dehydrogenase (PDH) complex that converts pyruvate to acetyl-coenzyme A (acetyl-CoA) (6). Thus, we speculated that S203 phosphorylation-mediated PGK1 mitochondrial translocation may be the core target for Ptc1 and Ptc2 to control metabolic processes. To determine the biological function of PGK1 in regulating mitochondrial pyruvate metabolism, we constructed a *pgk1* knockout strain (Δ*pgk1*) (Fig. S15A, S15C, and S15D). We quantified the contents of acetyl-CoA in the mitochondria and pyruvate in the cytoplasm. PGK1 depletion, similar to PGK1^S203A^, significantly enhanced the conversion of pyruvate to acetyl-CoA (Fig. 8E and F). These effects were abrogated by the complemented expression of PGK1. In contrast, PGK1^S203D^ expression could not lead to acetyl-CoA production by converting pyruvate in the mitochondria, which leads to pyruvate accumulation in the cytoplasm (Fig. 8E and F). Acetyl-CoA, a precursor for aflatoxin synthesis, appears to be the first step in the aflatoxin biosynthesis pathway that is then used for the formation of the aflatoxin initiation unit hexanoate (46).

PGK1^S203D^ expression also failed to induce the production of aflatoxin AFB_1_ compared with the WT, complementary strain, and PGK1^S203A^ (Fig. 8G and H). In contrast, PGK1 depletion significantly enhanced aflatoxin synthesis. The phosphorylation of mitochondrial PGK1, therefore, suppresses mitochondrial acetyl-CoA production by attenuating the mitochondrial pyruvate metabolism, inhibiting aflatoxin biosynthesis in *A. flavus.*

### Effects of PGK1 dephosphorylation on autophagy

**Fig 9:**
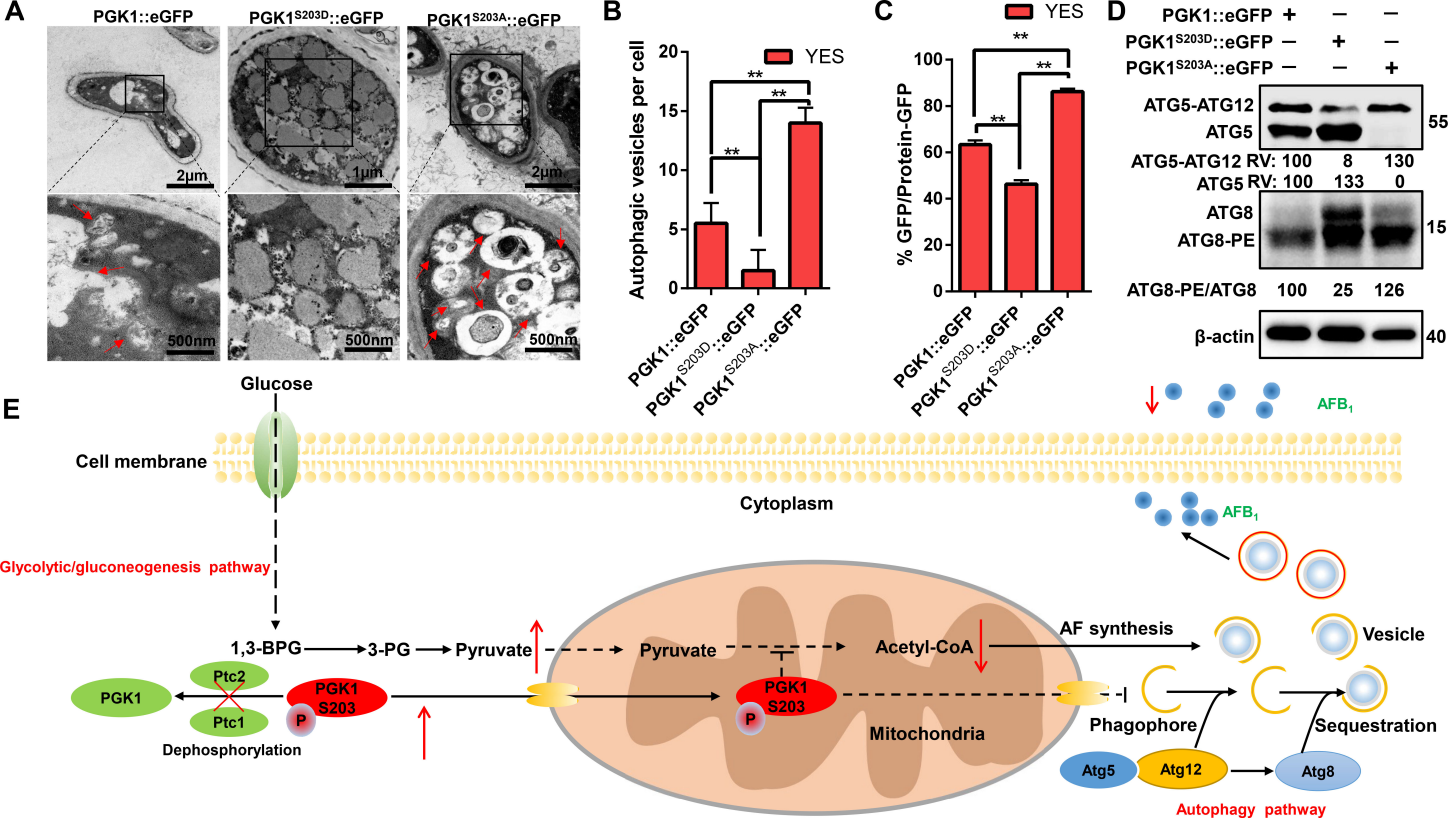
Dephosphorylation of PGK1 S203 promotes autophagy in *A. flavus.* (**A**) Representative TEM images of autophagosomes in PGK1::eGFP, PGK1S203D::eGFP, and PGK1S203A::eGFP strains for 5 days. Red arrow, autophagic vesicles; scale bar: 500 nm. (**B**) Quantification of an average number of autophagic vesicles per cell. Error bars represent SD and statistical significance was determined by ANOVA (Dunnett *t*-test). ** presents *P* ≤  0.01. (**C**) The ratio of free GFP to the total GFP signal in three independent experiments from PGK1::eGFP, PGK1^S203D^::eGFP, and PGK1^S203A^::eGFP, as described in Fig. 8B (left panel). The β-actin served as loading control for standardization of each relevant sample. Error bars represents SD and statistical significance was determined by ANOVA (Dunnett *t*-test). ** present *P* ≤  0.01. (**D**) PGK1::eGFP, PGK1S203D::eGFP, and PGK1S203A::eGFP were analyzed using immunoblotting. The relative expression levels of Atg5, Atg5-Atg12, and Atg8-PE/Atg8 were normalized by β-actin for each group. The intensity of fusion protein in WT was defined as 100. (**E**) Proposed regulation of aflatoxin production via Ptc1 and Ptc2 phosphatases. Ptc1 and Ptc2 are involved in aflatoxin biosynthesis and autophagy induction by affecting mitochondria-translocated PGK1. The phosphorylated S203 of PGK1 negatively regulates synthesis of acetyl-CoA in mitochondria and autophagy. Broken arrows represent inhibited directions or reactions. The red cross indicates deficiency.

The PPI network of Ptc1 and Ptc2 showed that PGK1 in the glycolysis/gluconeogenesis pathway interlinks with the phagosome pathway (Fig. 7D). We, therefore, investigated whether PGK1 S203 phosphorylation could result in excessive autophagy induction (Fig. S16A and B). Autophagic fluorescence was significantly increased in the Δ*pgk1* and S203A mutants. In contrast, the MDC fluorescence of PGK1 S203D, the constitutive phosphorylation mutant of PGK1, was the weakest among all the other strains (Fig. S16B). An electron microscope ultrastructural analysis showed that the number of autophagic vesicles was significantly lower in PGK1 S203D than in the WT and PGK1 S203A mutants (Fig. 9A and B).

In yeast, the degradation of PGK1-GFP followed by immunoblotting reliably quantifies autophagy (47). Thus, we assessed whether the degradation of PGK1-GFP quantifies autophagy in *A. flavus* (Fig. S16C and D). The ratio of free GFP to PGK1-GFP significantly increased in PGK1::eGFP cells after treatment with rapamycin or MMS. In line with the autophagic vesicle staining results in the PGK1::eGFP strains stained with MDC (Fig. S16E), the autophagic fluorescence was significantly enhanced under the same autophagy-induced conditions, indicating that PGK1 degradation in *A. flavus* can be used to quantify autophagy.

We measured the protein degradation of PGK1-GFP in the S203 point mutations (Fig. 8B and 9C). PGK1^S203A^::eGFP was significantly degraded compared to PGK1::eGFP and PGK1^S203D^::eGFP. To determine the effect of the S203 site on autophagic vesicle membrane formation, the Atg5 and Atg8 protein levels were assessed in *pgk1* deletion and PGK1 S203D/A point mutants. The protein levels of Atg5–Atg12 and the ratio of Atg8-PE/Atg8 were markedly decreased in PGK1 S203D compared to those in the WT and PGK1 S203A strains (Fig. 9D). Collectively, these data suggest that Ptc1 and Ptc2 participate in autophagy by dephosphorylating PGK1 S203 in *A. flavus*.

## DISCUSSION

We revealed that PP2C phosphatases affect growth, conidial formation, and carcinogenic aflatoxin production in *A. flavus*. The identification and functional study of PP2C phosphatase in *Saccharomyces cerevisiae* (Ptc1–7) are complete and in-depth (18, 48). The bioinformatic analysis fully identified seven PP2C phosphatases (Ptc1, Ptc2, Ptc3, Ptc4, Ptc5, Ptc6, and Ptc7) in the genome of *A. flavus* that are orthologs of those in *S. cerevisiae* (Ptc1–Ptc7). Similarity searches are effective for identifying homologous proteins. Proteins with statistically significant sequence similarity can be inferred to be homologous and have similar structural domains, which perform similar functions (49). However, identifying *A. flavus* phosphatase depends on the quality of the yeast PP2C phosphatase annotation, and may miss other homotypic phosphatases. Previous studies have described CaPtc8 protein to add to CaPtc1–7 as a new member of PP2C genes specific to *Candida albicans.* There are two families of proteins with similarity to *S. cerevisiae Ptc7* including a “typical” Ptc7s in all organisms and a Ptc7-related protein only present in the CTG clade (organisms that translate the CTG codon as serine instead of leucine). Thus, the identification of other fungal PP2C phosphatases also referred to the model fungus *S. cerevisiae*, including *Fusarium graminearum*, *Candida albicans*, *Fusarium oxysporum*, and *Botrytis cinerea* (23, 24, 50). The global analysis of *Aspergillus* spp. showed the presence of multiple PP2C proteins (18, 23). These identified PP2Cs phosphatases are similar to the seven PP2C protein sequences from *S. cerevisiae* (18). This indicates that the reference annotations of PP2C phosphatase of *S. cerevisiae* are comprehensive and reliable. More importantly, the number of phosphatases identified by *A. flavus* is consistent with other model fungi (18, 23, 50). Therefore, the identification of *A. flavus* PP2C phosphatase is accurate and reasonable.

Phenotypic evidence showed that only Ptc1 and Ptc2 are involved in growth morphology, conidial production, and aflatoxin biosynthesis in *A. flavus*, with Ptc2 performing more major functions. Based on these findings, we propose that the loss of Ptc1 and Ptc2 significantly increases the phosphorylation of PGK1. The phosphorylated PGK1 is then shuttled into the mitochondria, inhibiting mitochondrial metabolism, autophagy, and aflatoxin synthesis (Fig. 9E). Our study observed that Ptc1 and Ptc2 regulate conidial and sclerotia formation and aflatoxin synthesis, but Ptc2 was indispensable and dominant in *A. flavus*. In yeast, the transcriptional profiles of the *ptc1* mutants are significantly distinct from those of *ptc2–ptc5* mutants under standard conditions, although they may have overlapping functions (13, 15). For example, Ptc1, Ptc2, and Ptc3 phosphatases negatively regulate the Hog1 mitogen-activated protein (MAP) kinase through dephosphorylation under osmotic stress. Ptc2 and Ptc3 suppress HOG pathway activation significantly more than Ptc1 (14, 18). The different PP2Cs have slight differences in their functions. The Tm result indicates that the thermal stability of Ptc2 is higher than that of Ptc1. The stability of different phosphatases leads to the differences in enzyme activity, which is also a key factor affecting their biological functions (1, 51). The stability of proteins is closely related to their enzymatic activity (52). We found that the phosphatase activity of Ptc2 is also higher than that of Ptc1. Therefore, *ptc1* and *ptc2* may perform overlapping biofunctions in *A. flavus*, with *ptc2,* a stable and high enzyme activity phosphatase*,* playing a more major role in growth, development, and aflatoxin synthesis.

PP2Cs act as single-subunit enzymes that bind Mn^2+^ or Mg^2+^ through conserved catalytic core region (29). The catalytic domain contains a highly conserved metal-coordinating Asp residue essential for substrate dephosphorylation (53, 54). To study the catalytic cores of Ptc1 and Ptc2, the highly conserved Asp residues (Asp-204, Asp-338, and Asp-442 of Ptc1 and Asp-43, Asp-120, and Asp-212 of Ptc2) coordinating the metal ions in the center were site-mutated *in vitro* and *in vivo*. These Asp site mutations can affect the stability and conformation in Ptc1 and Ptc2 phosphatase. The closeness of the chelation between the Asp and metal ions is crucial to maintain the structural stability of the PP2C phosphatase (55). However, not all Asp residues can affect the catalytic activity to perform a function. Phosphatase activity tests *in vitro* showed that only Ptc1 D338A had unchanged enzymatic activity, whereas Ptc1 D338 *in vivo* affected sclerotia formation and aflatoxin production through site mutations in *A. flavus.* In human PPM isoforms, the metal-coordinating Asp residues of the PPMs have another function, aside from substrate dephosphorylation (54). Hence, we speculate that the Asp of the conserved catalytic core plays various roles in regulating biological processes *in vivo,* not limited to simple phosphatase catalytic functions. Phosphatase conformational change caused by Asp mutation can affect different substrate recognition (55, 56).

The Asp point mutations at different positions in Ptc2 *in vitro* led to the inconsistent downregulations of phosphatase activity in the following order: WT > D43A > D212A > D120A. D120A had a greater influence than D212A or D43A on the biological functions of Ptc2, such as inhibiting of fungal development, aflatoxin synthesis, and pathogenicity. The discrepancy in enzymatic activity caused by the Asp mutations in different sites can be attributed to coordination being loosely bound by divalent metals in the active center, or the so-called “volatile” metals in the PPMs (54). Thus, although the catalytic sites of the PP2C homologs comprise a high degree of structural conservation in *A. flavus*, the diversity in fixity of metal may result in different activities among the enzymes (54). Therefore, the conserved metal ion-coordinating Asp residues are necessary for the biological function of Ptc1 and Ptc2 in *A. flavus.* Msg5 and Yvh1 contribute to growth, development, aflatoxin biosynthesis, pathogenicity, and Fus3 dephosphorylation in *A. flavus* (37); however, Msg5 is a negative regulator, indicating that members of the same phosphatase family can exhibit different intensities in regulating biological processes. We speculate that the main reason for the different distributions at the regulatory level is the different activities of the phosphatases.

As a potential and specific inhibitor, sanguinarine is attractive, and can target the PP2C type domain of phosphatase for inactivation (31, 56). The hyper-activation/dysfunction of PP2Cs is linked to the virulence and growth of filamentous pathogenic fungi (25, 27 – 29). This is important practically as sanguinarine’s inhibition to plant PP2C improves its efficacy against other PP2Cs, and has potential use in agriculture for crop protection against stresses, pathogens, and herbivores (33, 57). The sanguinarine targeted inhibition of MoMPG1 impaired the appressorium-mediated penetration and pathogenicity of *M. oryzae* (58). In mammalian cells, sanguinarine is described as inhibitor for dual-specific PP2Cs between PP2Cα and mitogen-activated protein phosphatase-1 (31). Therefore, *A. flavus* Ptc1 and Ptc2 may be the key phosphatases for sanguinarine to perform inhibitory functions. The available catalytic core region of Ptc1 and Ptc2 provided valuable information for molecular modeling to the fungal-specific inhibitor. Because sanguinarine inhibition mimics the deletion of either Ptc1 or Ptc2, studying their catalytic mechanisms is very important for developing it as a therapeutic target for controlling aflatoxin production and protecting crops.

IP-MS is a preferred method for exploring physiologically relevant protein-protein interactions (59). Both Ptc1- and Ptc2-interacting proteins are mainly located in the cytoplasmic component, and some are also distributed in other organelles such as nucleus and mitochondria. The interactions of Ptc1 and Ptc2 with these target proteins are classified into physiological and non-physiological. For the physiological interactions, we suspect that Ptc1 and Ptc2 perform biological functions by translocation between different organelles. Ptc5 of *S. cerevisiae* in the cytosol can translocate to peroxisomes (9). In this study, Ptc1 and Ptc2 were also translocated to the nuclei after the MMS treatment. However, under no treatment conditions, Ptc1 and Ptc2 were distributed in the cytoplasmic. These nuclear proteins identified by IP-MS may represent non-physiological interactions, such as histone H3, H1, and H4. In immunoprecipitation process, mycelium proteins were lysed in radioimmunoprecipitation assay (RIPA) buffer. The membrane boundary was destroyed, allowing the mixing of proteins from different compartments after cell lysis. This is the primary source of non-physiological interactions (false-positive interactions) during the purification (59). These histones, which regulate a wide range of cellular processes, have abundant pS/pT sites (60, 61). This may be why these proteins are recognized by the Ser/Thr protein phosphatases Ptc1 and Ptc2 in non-physiological conditions and identified by IP-MS. To address these limitations, the use of protein proximity labeling techniques and biochemical co-fractionation of native protein complexes, combined with chemical crosslinking with MS, could provide a strategy to study protein-protein interactions in a living cell context and a near-native physiological context, respectively (62).

Proteins interacting with Ptc1 and Ptc2 regulate glycolysis/gluconeogenesis, carbon metabolism, amino acid biosynthesis, and the phagosome pathway. However, Ptc2-interacting proteins are uniquely enriched in fatty acid metabolites, 2-oxocarboxylic acid metabolism, and fructose and mannose metabolism as opposed to Ptc1-interacting proteins, which indicates the molecular mechanism by which Ptc2 plays a more dominant role in metabolism. Moreover, compared with the homologous family members Ptc5p and Ptc7p, Ptc6p regulated the entry of glycolysis-derived pyruvate into the TCA cycle in *S. cerevisiae* (63). Although Ptc1 and Ptc2 also exhibit some different functions in *A. flavus*, they have conserved active sites and share similar catalytic mechanisms to regulate co-substrate proteins. We found that Ptc1 and Ptc2 perform similar functions regulating aflatoxin synthesis and autophagy by dephosphorylating PGK1 S203 in *A. flavus*. In agreement with Ptc2 and Ptc3 in *S. cerevisiae,* the two phosphatases play redundant roles in autophagy by interacting with Atg1 and Atg13 and dephosphorylating them (20). Supporting this, our work found that these two enzymes have many common interacting substrates which are dephosphorylated to regulate the same function, such as Slt2 and PGK1. Using the medium confidence score obtained from the STRING database (interaction score ≥0.4), Ptc1 and Ptc2 were inferred to interact for regulating various biological processes, such as metabolism and autophagy in *A. flavus*. The interaction between Ptc1 and Ptc2 in fission yeast and *S. cerevisiae* was confirmed using genetic interference assays (42, 64, 65). Our data suggested that Ptc1 and Ptc2 co-regulate various substrates to perform biological functions. Recently, phosphatases have been used as co-sites on MAPKs in docking interactions, which are different from transient enzyme-substrate interactions (66). For example, Ptc1 does not directly bind to Hog1; however, the specificity of Ptc1 to Hog1, combined with the small adaptor protein Nbp2, was determined based on a docking interaction in yeast (67). Through docking interactions, Hog1 is jointly dephosphorylated by Ptc1, Ptc2, and Ptc3 (67). However, according to our list of interacting proteins, the interaction between Ptc1 and Ptc2 in *A. flavus* is not identified. We speculate that their interactions may be weak or indirectly given.

Autophagy is a conserved catabolic homeostasis process at the cell center (68). The induction of autophagy promotes the maturation of phagosomes, which are involved in protein degradation and regulating cellular metabolism (69, 70). The eGFP tag of Ptc1 and Ptc2 is cleaved from the fusion protein, primarily due to their involvement in the autophagic process (20). Fusion proteins undergo degradation in the autolysosome, resulting in the retention of the stable GFP tag (71, 72). The cleaved GFP tags become inactivated and their fluorescence is quenched, leading to a diffuse distribution with low brightness after autophagy induction (71, 73). Therefore, these cleaved GFP tags can aid in locating autophagy-related proteins through fluorescence, even if a significant portion of the eGFP tag is cleaved (74). We also demonstrated that Ptc1 and Ptc2 regulate autophagy and respond to autophagy-induced protein degradation. Ptc1- and Ptc2-interacting proteins are also co-enriched in phagosomes. Autophagosomes, which are double-membrane vesicles, play essential roles in the transport of aflatoxin in *A. flavus* and *A. parasiticus* (75, 76). In line with our findings, Ptc1 and Ptc2 redundantly promote autophagic vesicle formation, which controls the exocrine secretion of aflatoxin. However, a recent study suggested that Ptc2 and Ptc3 in yeast interact with the Atg1-Atg13 complex and are significantly degraded following rapamycin treatment but are autophagy-independent. Our PPI data indicated that Ptc1- and Ptc2-interacting proteins enriched in ribosomes are annotated as ubiquitin proteins (ubiquitin UbiA and UbiC) and the molecular chaperone Hsp70. In eukaryotes, a surveillance mechanism, known as ribosome-associated protein quality control, either facilitates substrate ubiquitylation and targeting for degradation or induces protein aggregation, which interlinks with the ubiquitin-proteasome system (77 – 79). Significant crosstalk between the ubiquitin-proteasome system and autophagy at multiple levels is present. One mechanism of ubiquitin-proteasome system (UPS)-rapamycin-induced autophagy is that changes in one protein decomposition pathway induce changes in the activity of another pathway (80, 81).

To identify the targets of Ptc1 and Ptc2 in secondary metabolism and autophagy processes, we assessed PGK1, which is involved in glycolysis/gluconeogenesis. Both Ptc1 and Ptc2 could bind to PGK1, leading to PGK1 phosphorylation at S203. Interestingly, the PGK1 ^S203A^ (non-phosphorylation) and PGK1 ^S203D^ (constitutive phosphorylation) mutants affected the PGK1 translocation into mitochondria. and its enzymatic activity. *In vivo* PGK1^S203A^ showed consistent results with *pgk1* deletion mutant in the conversion rate of pyruvate to acetyl-CoA and aflatoxin products in *A. flavus*. The extensive autophosphorylation of PGK1^S203^ (PGK1^S203D^ mutant) led to its translocation into the mitochondria, resulting in a moderate decrease in acetyl-CoA levels through the inhibition of pyruvate conversion. PGK1 kinase activity is activated by the phosphorylation of S203 (6, 82). Pten-induced kinase 1 (PINK1) regulates the binding of phosphorylated PGK1 at S203 to the translocase of the outer membrane of mitochondria (TOM) complex, which leads to mitochondrial translocation (6). In the mitochondria, PGK1 phosphorylation at S203 results in the repression of PDH-dependent pyruvate utilization and the increased cytosolic production of pyruvate (6, 82). Ptc1 and Ptc2 therefore target PGK1 S203 dephosphorylation, resulting in the integrated regulation of mitochondrial metabolism and aflatoxin synthesis. In addition, the dephosphorylation of PGK1 S203 significantly induced the formation of autophagic vesicles. Acetylation of PGK1 K388 phosphorylates Beclin1 S30 to regulate the initiation of autophagy, and affects cell metabolism, suggesting that these PTMs are important in autophagy and metabolism (83). Ptc1 and Ptc2 are involved in autophagy, mitochondrial metabolism, and aflatoxin synthesis by regulating PGK1 phosphorylation, indicating that the mutual regulation of autophagy and cell metabolism is integrated by PGK1 S203 phosphorylation. The dual roles of PGK1, which acts as both a glycolytic enzyme and protein kinase, are not ambivalent in functional control. The inhibition of autophagy impairs mitochondrial metabolism and affects the encapsulation of metabolites in double-membraned vesicles (84, 85). In *Aspergillus parasiticus* and *A. flavus*, vesicle formation in this delivery system is an indispensable step in the synthesis, compartmentalization, and export of aflatoxin (75, 76, 86). Based on our findings, autophagic vesicle formation is affected by PGK1 S203 phosphorylation, which indicates that Ptc1 and Ptc2 may regulate autophagy through PTMs of PGK1, thereby influencing aflatoxin biosynthesis.

Overall, we used the pathogenic fungus *A. flavus* as a model system to identify Ptc1 and Ptc2 as phosphatases for PGK1 and found that the activities of Ptc1 and Ptc2 contribute to mitochondrial metabolism, autophagy, and aflatoxin biosynthesis by promoting PGK1 dephosphorylation.

## MATERIALS AND METHODS

### Strains and culture conditions

The *A. flavus* strains used in this study are listed in Table S1. *Aspergillus flavus* was cultured on PDA for mycelial growth and conidiation as previously described (37). Sclerotia-inducing YPD medium was used to produce sclerotia (87). YES liquid medium was used to produce aflatoxin (88).

### Mutant strain construction

All *A. flavus* gene deletion mutants, complemented strains, and point mutants were generated using targeted PCR interchange based on a homologous recombination approach (37, 87). In brief, the upstream and downstream regions of the open reading frame (ORF) and *A. fumigatus pyrG* gene used as screening fragments were amplified and fused using specific primers. A high-quality purification product of the fusion fragment was transformed into *A. flavus* CA14 PTs. The complemented strains were constructed using a previously described protocol (37). The *pyrG* gene from a single-knockout mutant was exchanged using the complemented gene ORF in a resuscitation medium containing 2 mg/mL of 5-fluoroorotic acid. The screening gene *pyrG* was then reinserted into the above-mentioned strains to obtain the final complemented strain. To generate the *ptc1* and *ptc2* double-deficiency mutants, the pyrithiamine resistance marker *prtA*, which was amplified using the pPTRI vector (Takara), was used to replace the ORF of *ptc2* in Δ*ptc1* mutants. The pBabe-puro-mCherry-eGFP-Atg8 (Addgene, 22418) plasmid was reinserted into the protoplasts of *A. flavus* to generate the double-labeled autophagy fluorescence. Positive strains were screened with 100 mM phleomycin. All the strains were confirmed using PCR and RT-PCR (Fig. 3C and D). Site-directed mutants in the *A. flavus* strains were constructed using the fixed-point mutation primer method (89). The selected transformants were validated using PCR and DNA sequencing. Similarly, to produce protein-eGFP fusion strains, linker (10 amino acids) and eGFP tags were inserted between the ORF and terminator codon (TAA) (87). All strains were confirmed using PCR, and the fusion proteins were further analyzed by immunoblotting with mouse anti-GFP antibodies (Dia-An, China). The primer sequences used in this study are listed in Table S2.

### Gene cloning, protein expression, and purification

The full-length cDNA of *ptc1*, *ptc2*, and *PGK1* from *A. flavus* was cloned into the pET-21b vector (Novagen) to overexpress and purify proteins in *E. coli* according to the procedures described by the manufacturer. The primers used are listed in Table S2. The D204A, D338A, and D422A of Ptc2, D43A, D120A, and D212A of Ptc2, and S203A of PGK1 site-mutated proteins were prepared using the pET-21b/Ptc1 plasmids and pET-21b/Ptc2 with specific point mutation primers via overlapping extension PCR. The expression of the target protein from the pET-21b recombinant plasmid was induced in *E. coli* BL21 (DE3) cells following the addition of 0.5 mM isopropyl β-D-thiogalactoside (Biosharp, China). The detailed protein purification protocol used in this study has been previously described (90). The final purified proteins were visualized using a Coomassie Blue-stained gel following sodium dodecyl sulfate-polyacrylamide gel electrophoresis (SDS-PAGE). The WT and PGK1 S203A site-mutated proteins were used for an immunoblotting analysis.

### Analysis of phosphatase thermal stability

For thermal stability between WT of Ptc1/Ptc2 and their Asp mutants, the Nano-DSC instrument (Calorimetry Sciences Corp. Lindon, UT, USA) was used to measure the melting temperature of phosphatase in *vitro*. All protein samples (1.0 mg/mL) were purified and loaded into the sample CELL of the Nano-DSC System. Negative reference CELL was filled with clear bubbles of protein dissolution buffer (75 mM Tris-HCl, pH 7.5; 100 mM NaCl). The running program was then scanned using an increasing temperature from 25°C to 95°C with 1°C/min rate. The baseline value was determined by clear bubble protein dissolution buffer only. The curve fitting and data analysis were performed using DSC analysis software.

### Conidiation and sclerotia formation assays

To assess colony morphology and hyphal growth, approximately 5 µL of a 10^5^ spores mL^−1^ suspension of each strain was evenly coated on PDA medium, and then cultured at 37°C for 4 days in the dark. The colony diameters and conidia number were measured to analyze the differences in colony growth and conidial formation between the WT and all the mutants. Colony spores were collected from the flat surface of a PDA plate using 2 mL sterile water containing 0.5% Tween-20. For the conidiophore microscopic analysis, the PDA medium cultured at 37°C for 24 h was cut flat, transferred to a slide, and cultured in the dark at 37°C for 12 h. The conidiophores of all the strains were observed using a differential interference contrast microscope module. For the sclerotia analysis, each strain was inoculated and grown on YPD agar medium at 37°C in the dark for 7 days. Subsequently, 75% ethanol was used to wash off the hyphae on the surface of the medium. The fractions were then observed and counted. Each experiment was performed in triplicate with three replicates.

### Determination of aflatoxin production

All of the strains were evenly coated on YES liquid medium, peanuts, or maize seeds in the dark at 29°C for 7 days. To determine aflatoxin production, TLC was performed as previously described (91). Aflatoxin was extracted from the liquid media using chloroform extraction and resuspended in 100 µL of chloroform. The aflatoxin extraction samples were identified using TLC in a solvent system (chloroform:acetone = 9:1). The plates were then examined under a UV light at 365 nm. The resultant aflatoxin was quantitatively analyzed using ImageJ software.

### Seed pathogenicity assays

*Aspergillus flavus* infections in the peanuts and maize seeds were detected using a previously described method (92). Uniform peanuts and maize kernels were screened, and aseptic cleaning procedures were carried out using 0.05% sodium hypochlorite for 5 min, low-velocity vibration of sterile water for 10 s, 75% ethanol for 5 s, and sterile water for 5 s. Approximately 5 µL of a 10^5^ spores mL^−1^ suspension was soaked for 60 min and cultured in the dark at 29°C for 6 days. All infected seeds were transferred into a 50 mL centrifuge tube containing 15 mL of 0.05% Tween-20 in sterile water. Conidia and aflatoxin were collected and quantitatively analyzed as described above. The experiment was independently repeated three times.

### Quantitative real-time PCR analysis

*Aspergillus flavus* mycelia were collected and fragmented from the PDA, YES, and YPD media for 48 h. TRIzol reagent (Biomarker Technologies, China) was used to extract the total RNA according to a previously described method (93). cDNA was synthesized using RT-PCR with a First-Strand cDNA Synthesis Kit (TransGen Biotech, China). The specific primers are listed in Table S2 and were used to perform quantitative PCR with the above-mentioned cDNA as a template. The relative transcript levels of each gene were quantified using the 2^-ΔΔCt^ method (37), with β-actin as the endogenous standard. All the experiments were performed in triplicate.

### Phosphatase assays

Ptc1, Ptc2, and their site-directed mutated proteins were purified using a Ni^2+^ affinity column. The enzymatic activities of all the phosphatases after ultrafiltration were measured as previously described (11). In brief, 0.5 µg of phosphatase was added to 100 µL of reaction solution (containing 75 mM Tris [pH 7.6], 0.5 mM EDTA, 100 mM NaCl, and 5 mM p-nitrophenyl phosphate) supplemented with 10 mM MnCl_2_, CaCl_2_, or MgCl_2_. Absorbance data at 405 nm were acquired after 30 min using a MiniMax Imaging Cytometer (Molecular Devices, Sunnyvale, CA, USA). A standard curve was constructed using 4-nitrophenol to determine phosphatase activity. For the *in vivo* dephosphorylation assays, mycelial fragments of *A. flavus* were collected from the WT and mutant strains grown at 29°C and analyzed using an alkaline phosphatase assay kit (Solarbio, China).

### Subcellular fractionation

The mitochondrial and cytosolic fractions of the *A. flavus* hyphae were extracted using a Tissue Mitochondria Isolation Kit according to the manufacturer’s instructions (Beyotime Biotechnology, China). The fractions were used to analyze the pyruvate and acetyl-CoA contents. Cytosolic pyruvate and mitochondrial acetyl-CoA concentrations were measured using a pyruvate assay kit (Solarbio, China) and an acetyl-CoA fluorometric assay kit (Solarbio, China), respectively. The nucleus was extracted using a Nucleus Separation Extraction Kit (Solarbio, China). For the immunoblotting analyses, proteins from the mitochondrial fraction were disintegrated and isolated using RIPA buffer (Biosharp, China). After denaturing the proteins at 98°C for 5 min, 20 µg of protein in SDS loading buffer was analyzed using SDS-PAGE and immunoblotting.

### Inhibitor treatments and induction of autophagy

For the PP2C inhibition treatments, 1, 10, 50, and 100 µM sanguinarine chloride (Aladdin, China) diluted in dimethyl sulfoxide (DMSO) were added to PDA and YES liquid media. An equal amount of DMSO was used as a loading control. MMS (Macklin, China) at a final concentration of 0.02% and rapamycin (Solarbio, China) at a final concentration of 200 ng/mL were used to induce macro-autophagy in *A. flavus*, as previously described (94).

### Fluorescence analysis

To determine the subcellular localization of Ptc1, Ptc2, PGK1, and the PGK1 site-mutated proteins in *A. flavus* cells, GFP fusion protein strains were cultured in YES liquid media alone or with 0.02% MMS or 200 ng/mL of rapamycin. The hyphae were washed three times with phosphate-buffered saline. Hoechst 33842 (Beyotime Biotechnology, China) and MitoTracker Red CMXRos (Beyotime Biotechnology, China) were used for nuclear and mitochondrial staining, respectively. A mycelium (10 µL) was spotted on the middle of a 76 × 26 mm microscope slide and covered with an 18 × 18 mm coverslip. Microscopy was performed using a Nikon Ti-U microscope and Zeiss Image A.2 with different filter sets for GFP (Ex 488 nm, Em 525/565 nm), mCherry (Ex 552  nm, Em 600/650  nm), Hoechst (Ex 405 nm, Em 420/460 nm), and MitoTracker Red (Ex 510 nm, Em 580 nm). The images were merged using ImageJ. A 1.5% agarose mycelial pellet was imaged to observe the conidia using a single-plane bright field. To observe the autophagic vesicles, MDC (Beyotime Biotechnology, China) staining solution was used to stain the autophagosomes and then analyzed using filter sets for green fluorescence (Ex 335 nm, Em 512 nm).

### Transmission electron microscopy (TEM)

*Aspergillus flavus* mycelia were fixed with 2.5% glutaraldehyde in 0.1 M sodium cacodylate buffer at 4°C. A TEM analysis was performed as previously described (95). After being dehydrated in ethyl alcohol and propylene oxide, the precipitated fungal tissues and cells were embedded in Epon (Electron Microscopy Sciences, 14120). The embedded samples were cut into ultrathin 75 nm sections using an Ultracut UCT ultramicrotome (Leica Biosystems) equipped with a diamond knife comprising a 45° blade (Diatome). The samples were stained at 37°C using 2% (wt/vol) aqueous uranyl acetate (5 min) and Reynold’s Lead Citrate (3 min) and analyzed using an HT7700 transmission electron microscope (HITACHI, Tokyo, Japan).

### Western blotting analysis

The hyphae of all the strains were harvested by centrifugation after being grown. Proteins were extracted using RIPA buffer after adding 1 mM phenylmethylsulfonyl fluoride (PMSF) protease inhibitor (Biosharp, China) as previously described (76). For the *in vitro* dephosphorylation assays, 0.5 µg of purified PGK1 was incubated in reaction solution (75 mM Tris [pH 7.6], 0.5 mM EDTA, 100 mM NaCl, and 10 mM MgCl_2_) with 0.2 µg of Ptc1 or Ptc2 proteins for approximately 2 h and collected as previously described (11). All of the proteins were denatured for 5 min at 98°C and 3,000 r/min before being loaded and separated using 12% SDS-PAGE. The samples were then transferred onto polyvinylidene fluoride membranes (Millipore, USA) in a Bio-Rad electroblotting apparatus (Bio-Rad, USA). The final membranes were developed using Western Bright ECL (Advansta, USA) and visualized using the CLINX ChemiScope 6000 Exp (CLINX, China). The hybridization signals of the western blots were quantified using ImageJ software (NIH). The following antibodies were used in this study: anti-GFP (1:5,000, 2057R, Dia-An), anti-GAPDH (1:5,000, 60004–1-Ig, Proteintech), anti-β-actin (1:5,000, PTM-5018, PTM Biolabs), anti-Slt2 with p44/42 MAPK (Erk1/2) (1:5,000, Cell Signaling Technology, 9102), anti-phospho-Slt2 with phospho-p44/42 MAPK (Erk1/2) antibody (1:2,000, Cell Signaling Technology, 4377), anti-ATG5 (1:1,000, PTM-5868, PTM Biolabs), anti-Atg8 (1:1,000, PTM-6384, PTM Biolabs), anti-PGK1 (1:2,000, A14039, ABclonal), anti-TIMM10 (1:2,000, A4626, ABclonal), anti-H3 (1:2,000, 68345–1-Ig, Proteintech), anti-pan-phospho-Ser (1:2,000, ICP9806, ImmuneChem), anti-pan-phospho-Thr (1:2,000, ICP9807, ImmuneChem), anti-phospho-PGK1 S203 (1:200, SAB487P, Signalway Antibody), and goat anti-rabbit IgG(H+L) (1:5,000, SA00001-2, Proteintech).

### Co-immunoprecipitation and mass spectrometry

For the co-immunoprecipitation analysis in *A. flavus*, the mycelia of the WT as negative control, Ptc1::eGFP, and Ptc2::eGFP were ground in liquid nitrogen and ultrasonically crushed in ice-cold RIPA buffer. The mycelium protein supernatant was collected and blended with GFP Nanoab Mag Beads (IP06M, Everylab) overnight at 4°C to recruit the target interaction proteins through immunoprecipitation. The magnetic beads were collected and washed with cold immunoprecipitation wash buffer three to five times. The immunoprecipitated proteins were detected using 12% SDS-PAGE and immunoblotted with anti-GFP antibody or target protein-specific antibody as previously described (96). To prepare the immunoprecipitation samples for a mass spectrometry analysis, 30 µL of the concentrated samples was separated using 12% SDS-PAGE, and the gels were stained with silver. To identify the proteins of silver-stained adhesive strips as comprehensively as possible, we performed four biological replicates for IP-MS. After in-gel tryptic digestion, the peptides were obtained and detected using liquid chromatography coupled with tandem mass spectrometry on a Q-Exactive mass spectrometer (Thermo Fisher Scientific) as previously described (87). pFind and MAX Quant software (version 2.0) was used to analyze the data.

### Bioinformatic analysis

The identified interaction proteins were classified based on their annotated GO terms using the eggNOG-mapper web (http://eggnog-mapper.embl.de/) (97) and TBtools software (98). The GO and KEGG pathway enrichment analyses were performed using the TBtools and STRING database (https://string-db.org) (99). For enrichment analysis, a *P*-value or *Q*-value less than 0.05 represents enrichment significance. *P*-value was corrected by Benjamini-Hochberg method. Subcellular localization was predicted using BUSCA (100). The PPI network was constructed using the STRING database and Cytoscape v3.6.1 (http://www.cytoscape.org) (101). Protein sequence was analyzed using multisequence alignments in BLASTP and TBtools. Sequence alignments were also performed using the CLC and Jalview sequence viewer.

### Statistical analysis

Unless otherwise specified, the data represent the results of at least three independent biological replicates. The data are expressed as means ± SD. The statistical analyses were performed in GraphPad Prism 8.0 (GraphPad Software). The Shapiro-Wilk normality test is used to determine whether data sets are normally distributed. For data that have passed the test, Student’s *t*-test was used for comparing two means differences. Otherwise, the data sets were performed for Mann-Whitney test. When multiple groups were compared, one-way ANOVA and Dunnett-T *post hoc* test (*P* < 0.05) were used to evaluate the statistical significance of the results. Two-way ANOVA with Tukey’s *post hoc* test was used to compare data sets across multiple factors. * and ** indicate that *P*-values are less than 0.05 and 0.01, respectively. ns is defined as no significance.

## Data Availability

The IP-MS proteomics data have been uploaded to the public database ProteomeXchange Consortium (http://proteomecentral.proteomexchange.org) via iProX with the data set identifier PXD038079 (102, 103).

## SUPPLEMENTAL MATERIAL

Supplemental figures mbio.00977-23-s0001.docx Fig. S1 to S16 and captions for Tables S1 to S9.

Table S1 mbio.00977-23-s0002.xlsx Strains.

Table S2 mbio.00977-23-s0003.xlsx Primers.

Table S3 mbio.00977-23-s0004.xlsx Proteins identified in IP-MS experiments.

Table S4 mbio.00977-23-s0005.xlsx eggNOG-mapper protein annotations for identified proteins.

Table S5 mbio.00977-23-s0006.xlsx Ptc1 and Ptc2 interacting proteins.

Table S6 mbio.00977-23-s0007.xlsx Subcellular localization of identified proteins.

Table S7 mbio.00977-23-s0008.xlsx GO function enrichment analysis of identified proteins.

Table S8 mbio.00977-23-s0009.xlsx KEGG PATHWAT and COMPARTMENTS enrichment analysis of identified proteins.

Table S9 mbio.00977-23-s0010.xlsx PPI for identifying proteins from STRING database.

ASM does not own the copyrights to Supplemental Material that may be linked to, or accessed through, an article. The authors have granted ASM a non-exclusive, world-wide license to publish the Supplemental Material files. Please contact the corresponding author directly for reuse.
