# Supplementary material for: PP2C phosphatases Ptc1 and Ptc2 dephosphorylate PGK1 to regulate autophagy and aflatoxin synthesis in the pathogenic fungus Aspergillus flavus
Source: mBio. 2023 Sep 27;14(5):e00977-23. doi: 10.1128/mbio.00977-23 (PMC10653812; doi:10.1128/mbio.00977-23)
Supplement: Supplemental figures — Fig. S1 to S16 and captions for Tables S1 to S9. [file mbio.00977-23-s0001.docx]

**SUPPLEMENTARY MATERIALS**

**
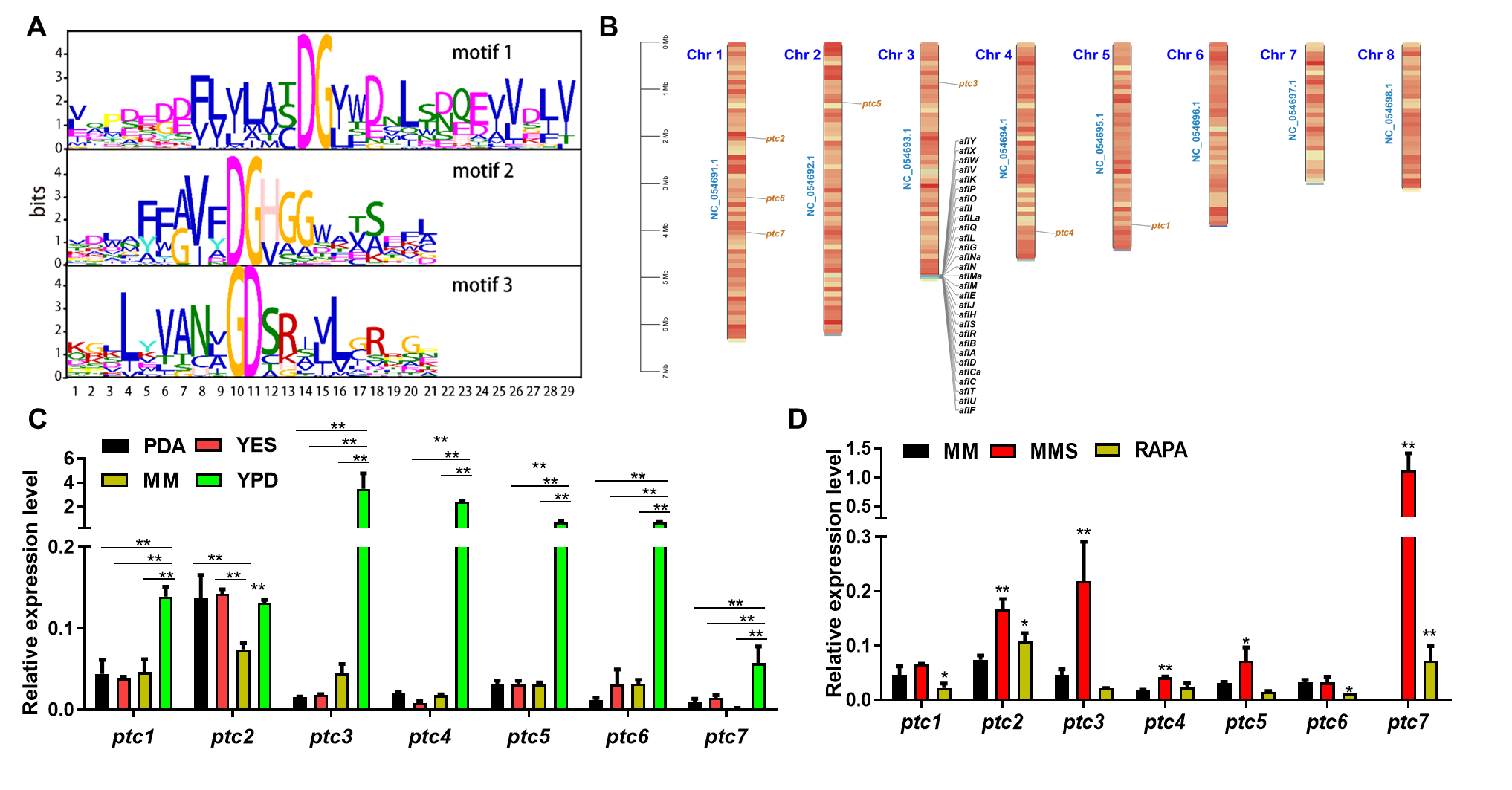
**

**Figure S1. Protein motifs, chromosome distribution positions, and expression profiles of PP2Cs in *A. flavus*.**  (A) The motifs 1, 2, and 3 from the PP2Cs were identified by MEME/MAST GUI Wrapper, as shown in Fig. 1A (middle panel). (B) Genes on the chromosome function map with PP2C genes in *A. flavus.* The positions of above genes are highlighted in red. Gene density profiles are shown by heatmaps. The chromosome map was generated using TBtools. (C) Expression patterns of PP2C family genes in *A. flavus* were tested by qRT-PCR in PDA, YES, MM, and YPD media, with *β-actin* as the reference gene. Data are mean ± standard deviation for 3 independent experiments, and statistical significance was assessed by one-way analysis of variance (ANOVA) and Dunnett-T post-hoc test. * *P ≤* 0.05; ** *P ≤* 0.01 (D) Expression patterns of PP2C genes from *A. flavus* in mycelial growth (MM), 0.02% MMS, and 200 ng/mL of RAPA (specific inhibitor of TOR). Data are presented with *β-actin* as a reference gene. Mean ± standard deviation for 3 independent experiments. Statistical analyses were performed by a one-way ANOVA followed by Dunnett-T post-hoc test. * *P ≤* 0.05; ** *P ≤* 0.01.


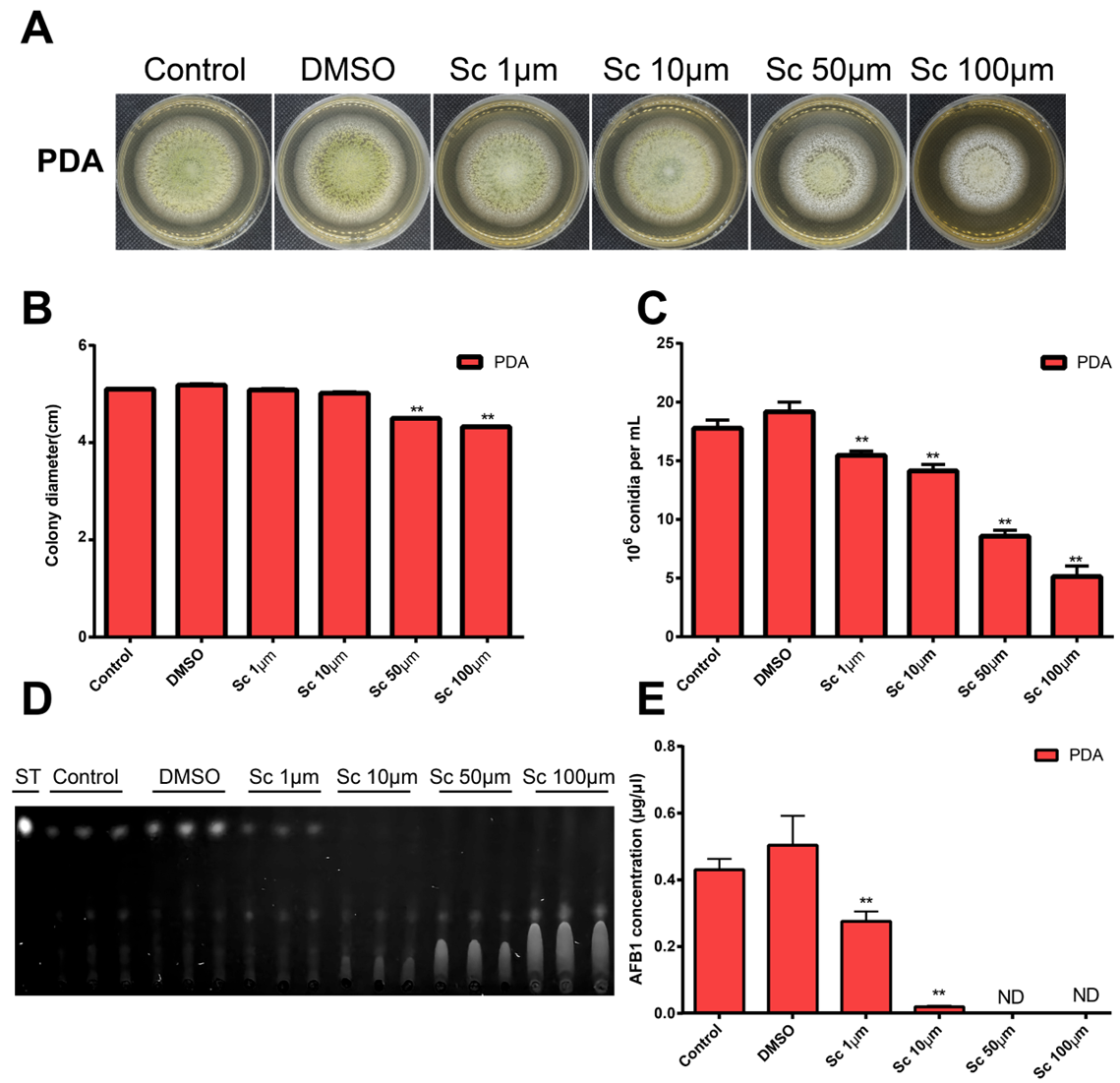


**Figure S2. Effects of the inhibitor sanguinarine on development and production of aflatoxin of *A. flavus*.** (A) Colony morphology with different concentrations of inhibitor sanguinarine on PDA media. (B–C) Colony diameters and conidia numbers produced by strains with different concentrations of sanguinarine on PDA media. Error bars represent SD and statistical analyses were performed by a one-way ANOVA followed by Dunnett-T post-hoc test. ** above bars represent significantly-different results (*P* ≤ 0.01). (D) Effects of treatment with different concentrations of sanguinarine on aflatoxin biosynthesis in *A. flavus.* (E) Quantitative analysis of aflatoxin production as in (D). The concentrations of sanguinarine used were 0 μm (equal-volume DMSO), 1 μm, 10 μm, 50 μm, and 100 μm. Sc represents inhibitor sanguine. Error bars represent SD and statistical analyses were performed by a one-way ANOVA followed by Dunnett-T post-hoc test. ** above bars represents significantly-different results (*P ≤* 0.01).


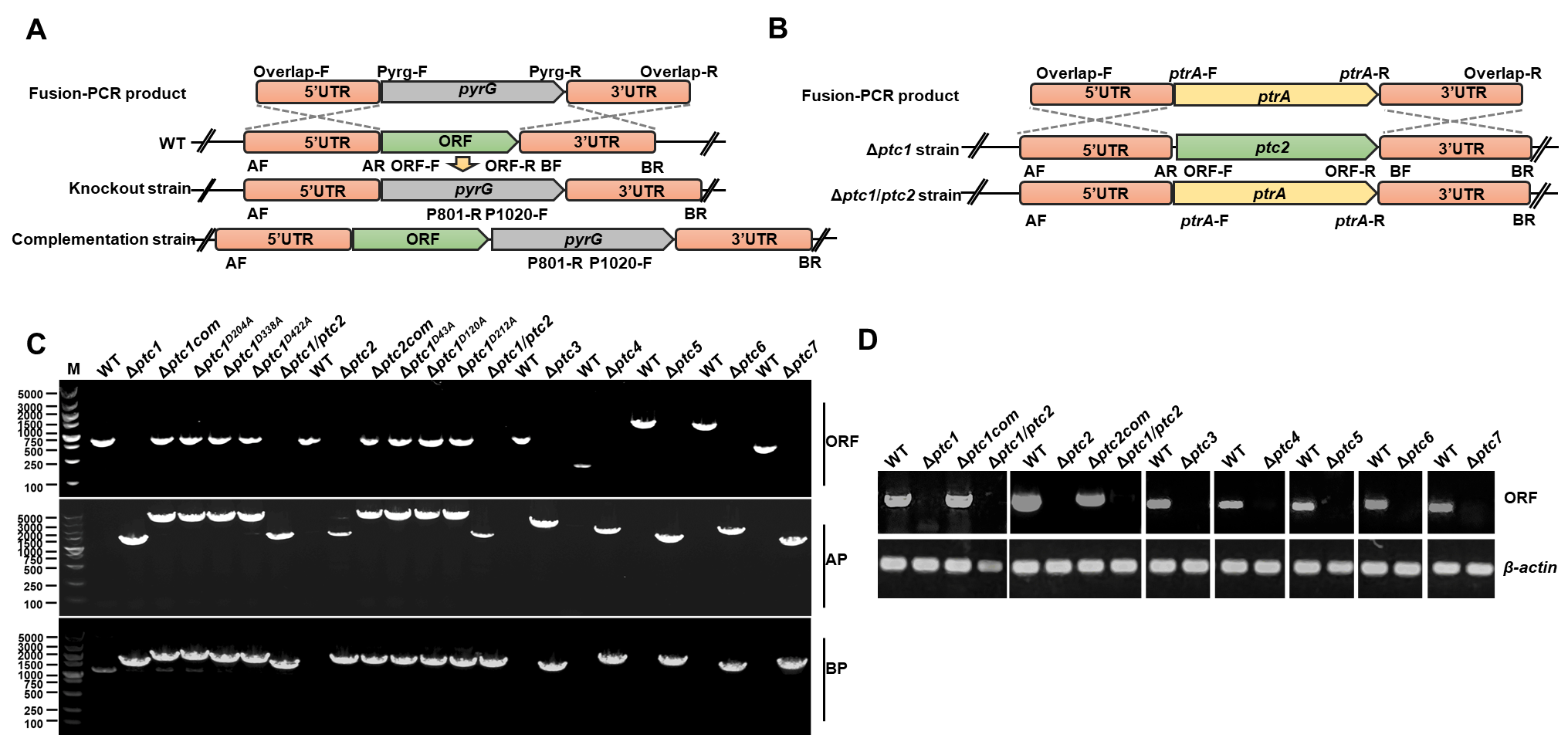


**Figure S3.** **Construction of PP2C mutants in *A. flavus*.** (A) Schematic showing the construction of PP2C knockout mutants generated using homologous recombination. The *Pyrg* cassette was inserted into CA14. The ORFs of PP2Cs were replaced, which led to deletion of the genes. (B) Schematic showing construction of *ptc1* and *ptc2* dual-knockout mutants generated using homologous recombination. The *ptrA* cassette was inserted into Δ*ptc1* to replace *ptc2* ORF. (C) Knockout, complement, and site point mutations of *ptc1*–*ptc7* were validated by PCR in *A. flavus.* AP represents the upstream region of the strain detected using these gene AF and P-801R primers; BP represents the downstream region of the strain detected using these gene BR and P-1020F primers; ORF region was detected using ORF detection primers (gene-D-F/R). (D) Knockout verification of genes (panel C) by RT-PCR using their RNA in *A. flavus* and *β-actin* as loading control.

**
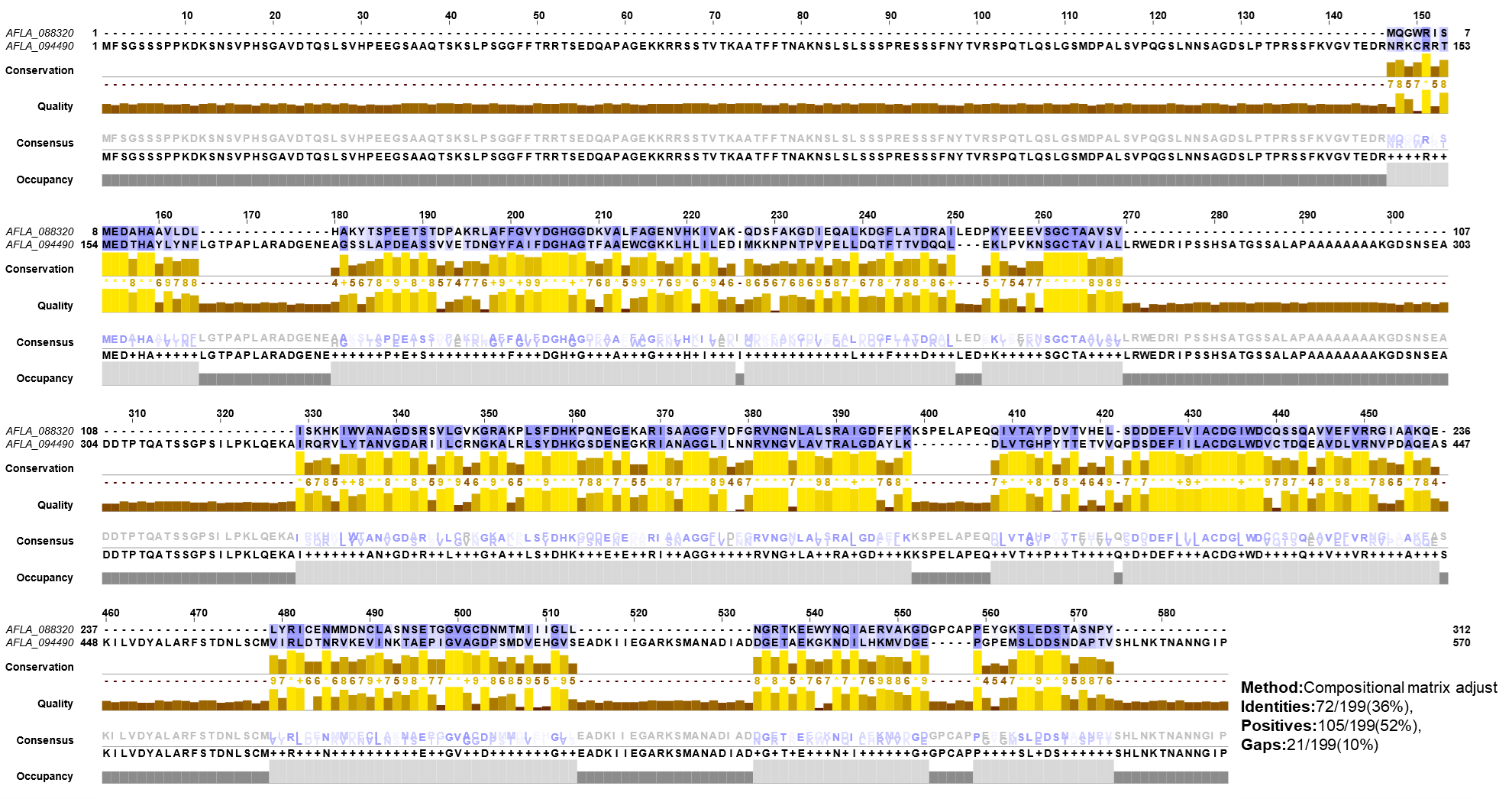
**

**Figure S4. Sequence comparison of PP2C** **protein phosphatase Ptc1 and Ptc2 in *A. flavus*.** The protein similar sequences are aligned and shaded with Jalview tool. The amino acid frequencies of conserved amino acid residues are shown in bar and Sequence Logo.


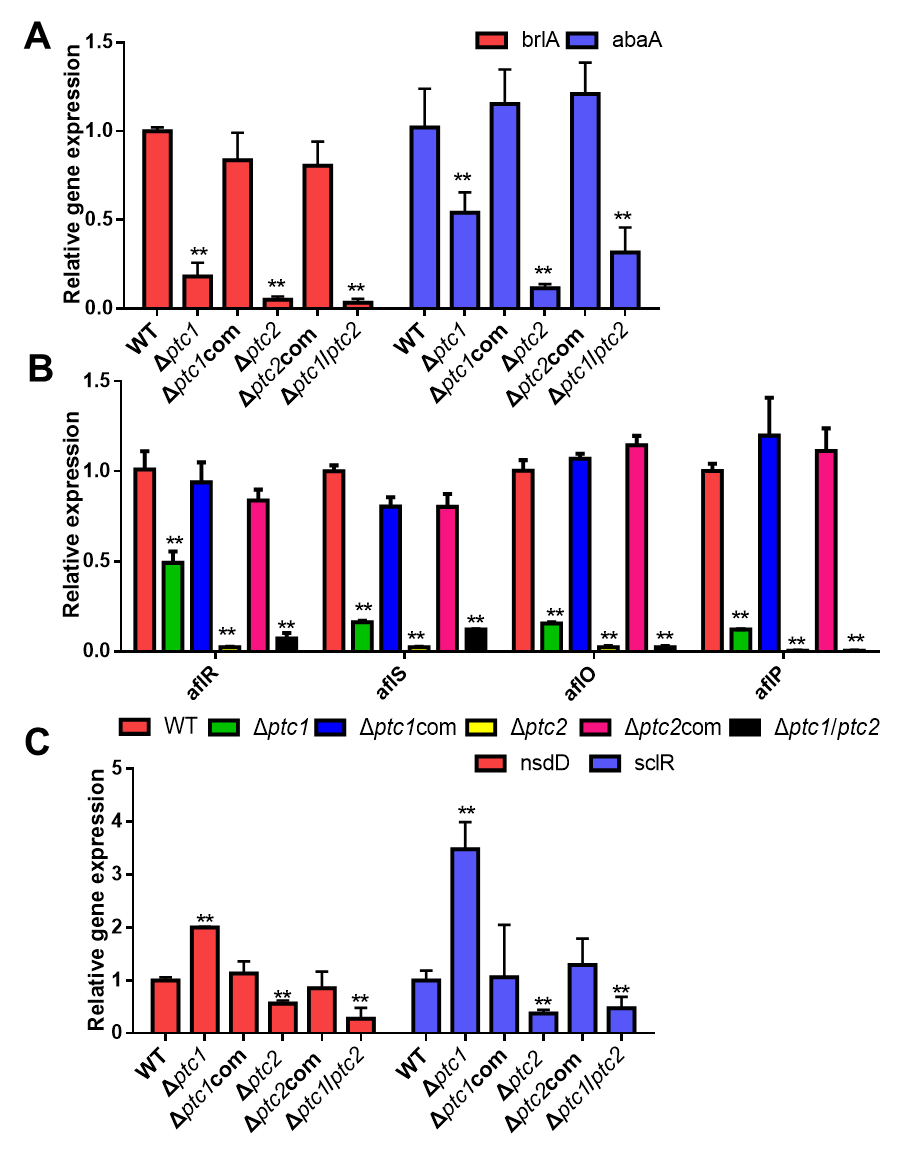


**Figure S5. Transcriptional levels of conidia-, sclerotia formation-, and aflatoxin cluster-related genes in the WT and gene-deleted mutants (*ptc1* and *ptc2*) in *A. flavus.*** (A) Transcriptional levels of the conidia-related genes *brlA* and *abaA*. Error bars represent SD and experiments were performed with four biological replicates for each strain and were repeated three times. Statistical analyses were performed by a one-way ANOVA followed by Dunnett-T post-hoc test. * and ** above the bars represent significantly different results (**P* ≤ 0.05, ***P* ≤ 0.01). The experiments were performed with four biological replicates for each strain and were repeated three times. (B) Relative expression levels of aflatoxin bio-synthesis regulatory and structural genes *aflR, aflS, aflO* and *aflP.* (C) Transcriptional levels of sclerotia-specific genes *nsdD* and *sclR*. Error bars represent SD and experiments were performed with four biological replicates for each strain and were repeated three times. Statistical analyses were performed by a one-way ANOVA followed by Dunnett-T post-hoc test. **P* ≤ 0.05 and ***P* ≤ 0.01


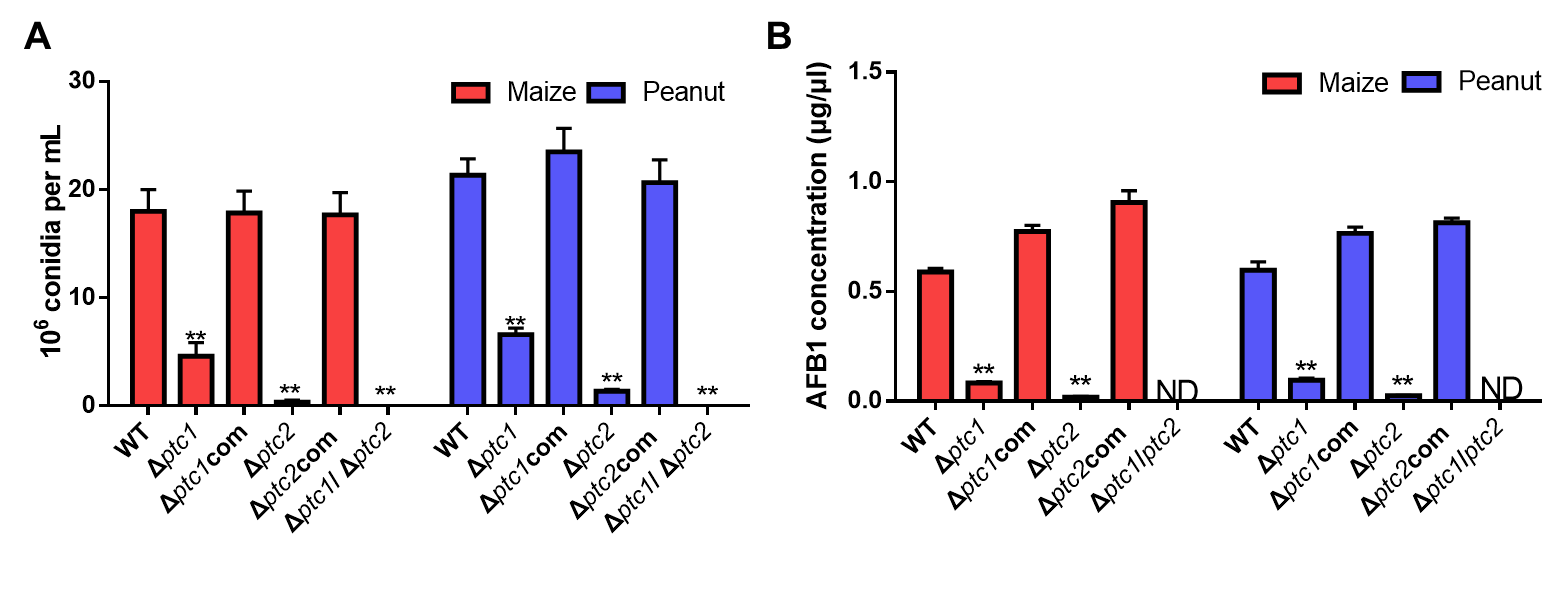


**Figure S6.** **Relative quantification of spore and aflatoxin production in infected maize and peanuts**. (A) Quantitative analysis of conidia production in infected seeds as described in Fig. 2H. Error bars represent SD and statistical analyses were performed by a one-way ANOVA followed by Dunnett-T post-hoc test. ** above bars represent significantly-different results (*P* ≤ 0.01). (B) Quantitative analysis of aflatoxin production as described in Fig. 2I. Error bars represent SD and statistical analyses were performed by a one-way ANOVA followed by Dunnett-T post-hoc test. (**P* ≤ 0.05, ***P* ≤ 0.01).


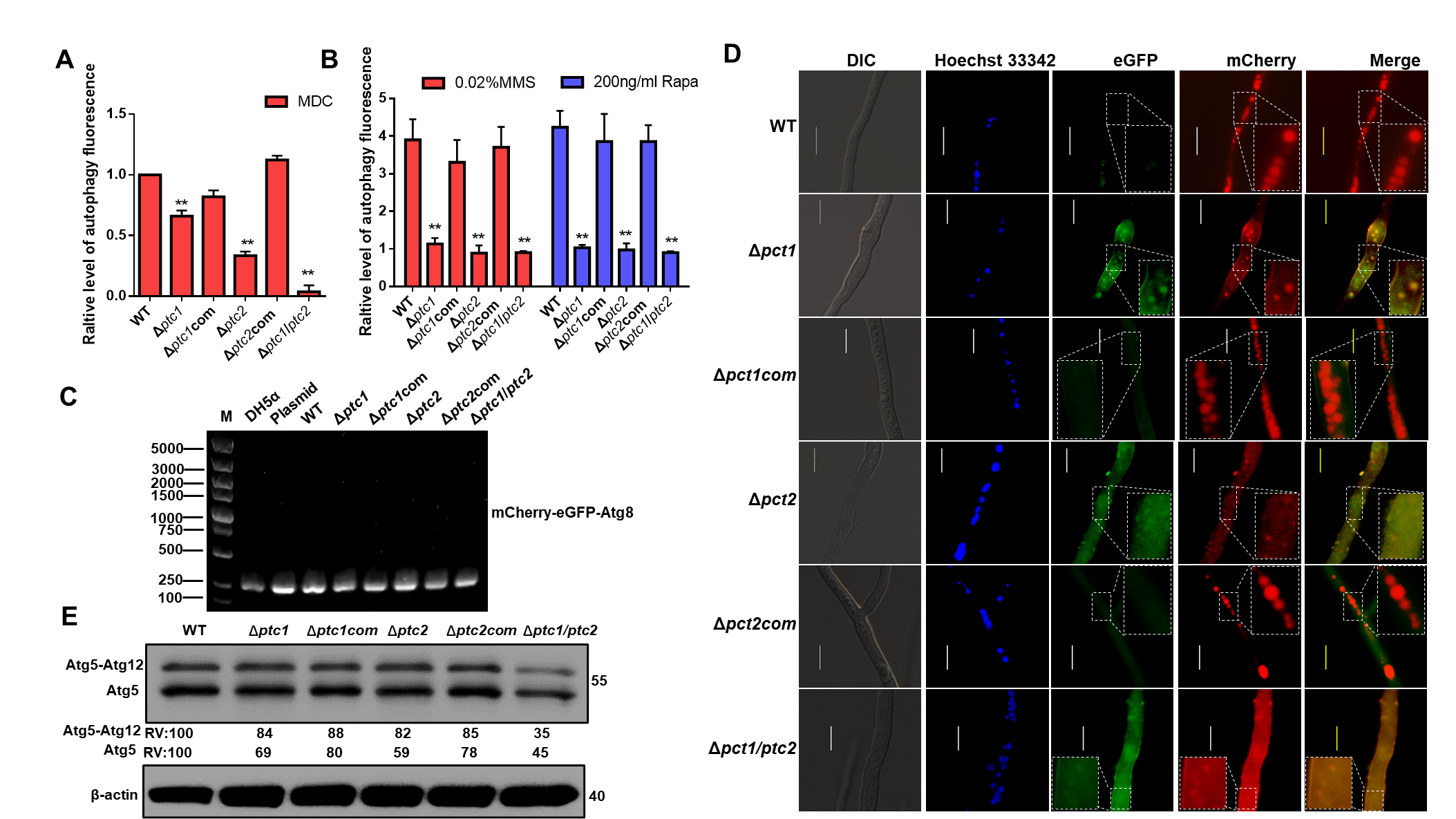


**Figure S7.** **Role of Ptc1 and Ptc2 in the formation of autophagic vesicles in *A. flavus*.** (A) Fluorescence intensity of the MDC stains in the WT, Δ*ptc1*, Δ*ptc1*com, Δ*ptc2*, Δ*ptc2*com, and Δ*ptc1*/Δ*ptc2* strains was measured on YES media using fluorescence microplate reader (Ex/Em = 335/518 nm). Mean ± standard deviation for 3 independent experiments. Statistical analyses were performed by a one-way ANOVA followed by Dunnett-T post-hoc test. (***P* ≤ 0.01). (B). Fluorescence intensity in the strains was determined after treatment with rapamycin or MMS. Mean ± standard deviation for 3 independent experiments. Statistical analyses were performed by a one-way ANOVA followed by Dunnett-T post-hoc test. (***P* ≤ 0.01 and ND represents not detected). (C) The WT, Δ*ptc1*, Δ*ptc1*com, Δ*ptc2*, Δ*ptc2*com and Δ*ptc1/*Δ*ptc2* expressing mCherry-eGFP-Atg8 strains were validated by PCR in *A. flavus*. *Escherichia coli* DH5α and plasmid mCherry-eGFP-Atg8 were used as positive controls. (D) The GFP and mCherry fluorescence of WT, Δ*ptc1*, Δ*ptc1*com, Δ*ptc2*, Δ*ptc2*com and Δ*ptc1*/Δ*ptc2* expressing mCherry-eGFP-Atg8 strains were observed in *A. flavus*. Representative images of GFP and mCherry fluorescence appear in YES medium for 48 h from vegetative hypha. Autophagosomes (merge: yellow) and autolysosomes (merge: red) appear in *A. flavus*. The nucleus was stained with Hoechst 33342. Scale bars, 10 μm. (E) The WT, Δ*ptc1*, Δ*ptc1*com, Δ*ptc2*, Δ*ptc2*com and Δ*ptc1*/Δ*ptc2* strains were analyzed using immunoblotting. Atg5 and Atg5-Atg12 levels were quantified. The intensity of fusion protein in WT was defined as 100. β-actin was used as a loading control.


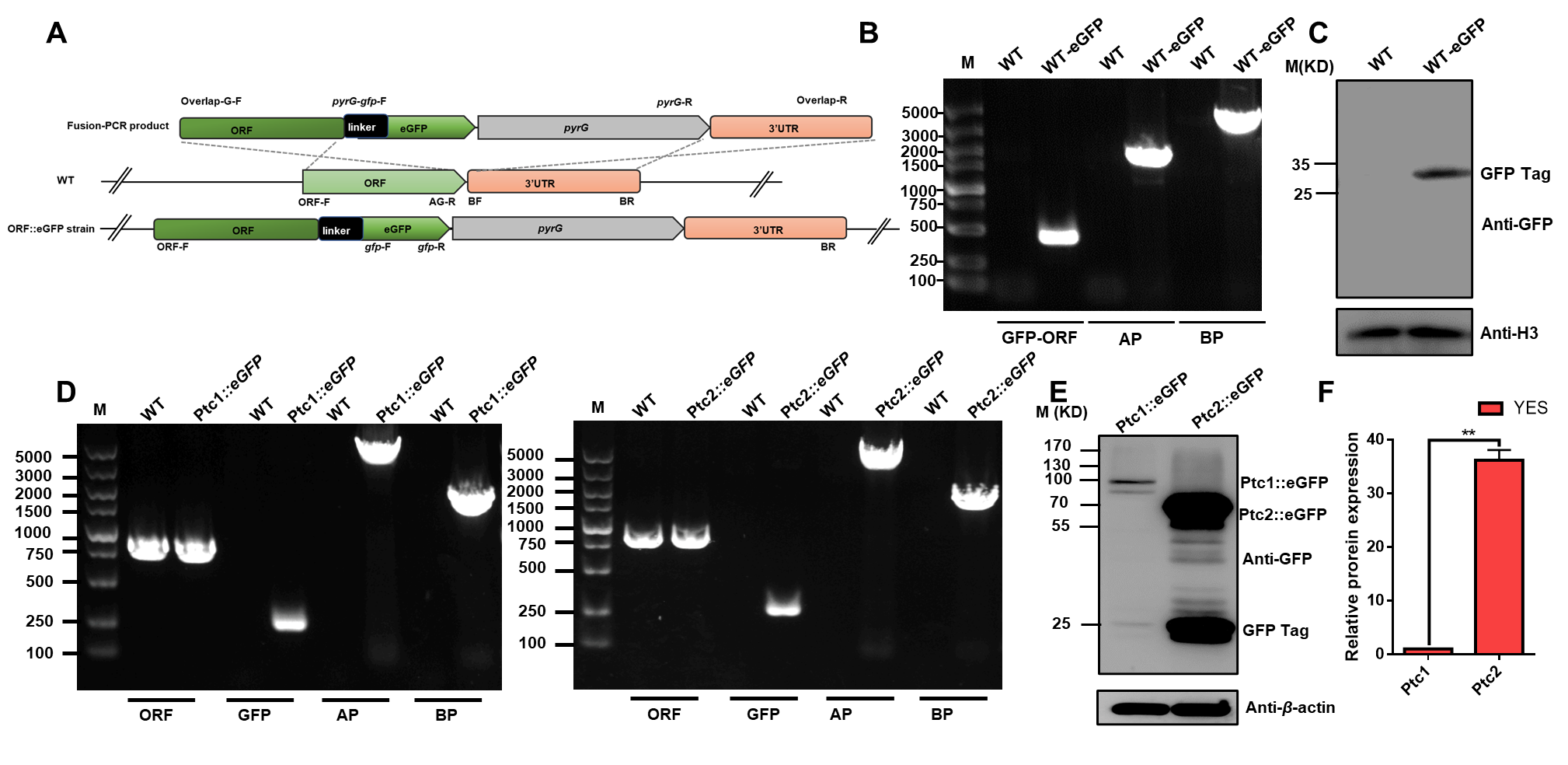


**Figure S8.** **Construction of Ptc1::eGFP and Ptc2::eGFP.** (A) Gene replacement strategy for the construction of Ptc1::eGFP and Ptc2::eGFP fusion protein strains. The black box represents a 10-amino-acid linker inserted between the protein and eGFP to ensure they did not affect each other. (B) WT-eGFP strains were verified by PCR using gDNA as a template. AP represents the upstream region of the strain detected using GFP-AF and P-801R primers; BP represents the downstream region of the strain detected using GFP- BR and P-1020F primers; GFP -ORF region was detected using ORF detection primers (GFP-D-F/R). (C) Western blotting was performed to detect the protein levels of GFP tag in WT-eGFP strain in PDA, with histone H3 as the standard reference. WT strains as negative control. (D) Ptc1::eGFP and Ptc2::eGFP strains were verified by PCR using gDNA as a template. AP represents the upstream region of the strain detected using *ptc1* or *ptc2* AF and P-801R primers; BP represents the downstream region of the strain detected using *ptc1* or *ptc2* BR and P-1020F primers; ORF region was detected using ORF detection primers (*ptc1* or *ptc2*-D-F/R). (E) Western blotting was performed to detect the protein levels of Ptc1 and Ptc2 in Ptc1::eGFP and Ptc2::eGFP in PDA, with β-actin as the standard reference. (F) Quantification of the levels of Ptc1 and Ptc2 as described in (E). Error bars represent SD. Student’s t test was used when comparing two means for differences. (***P* ≤ 0.01).

**
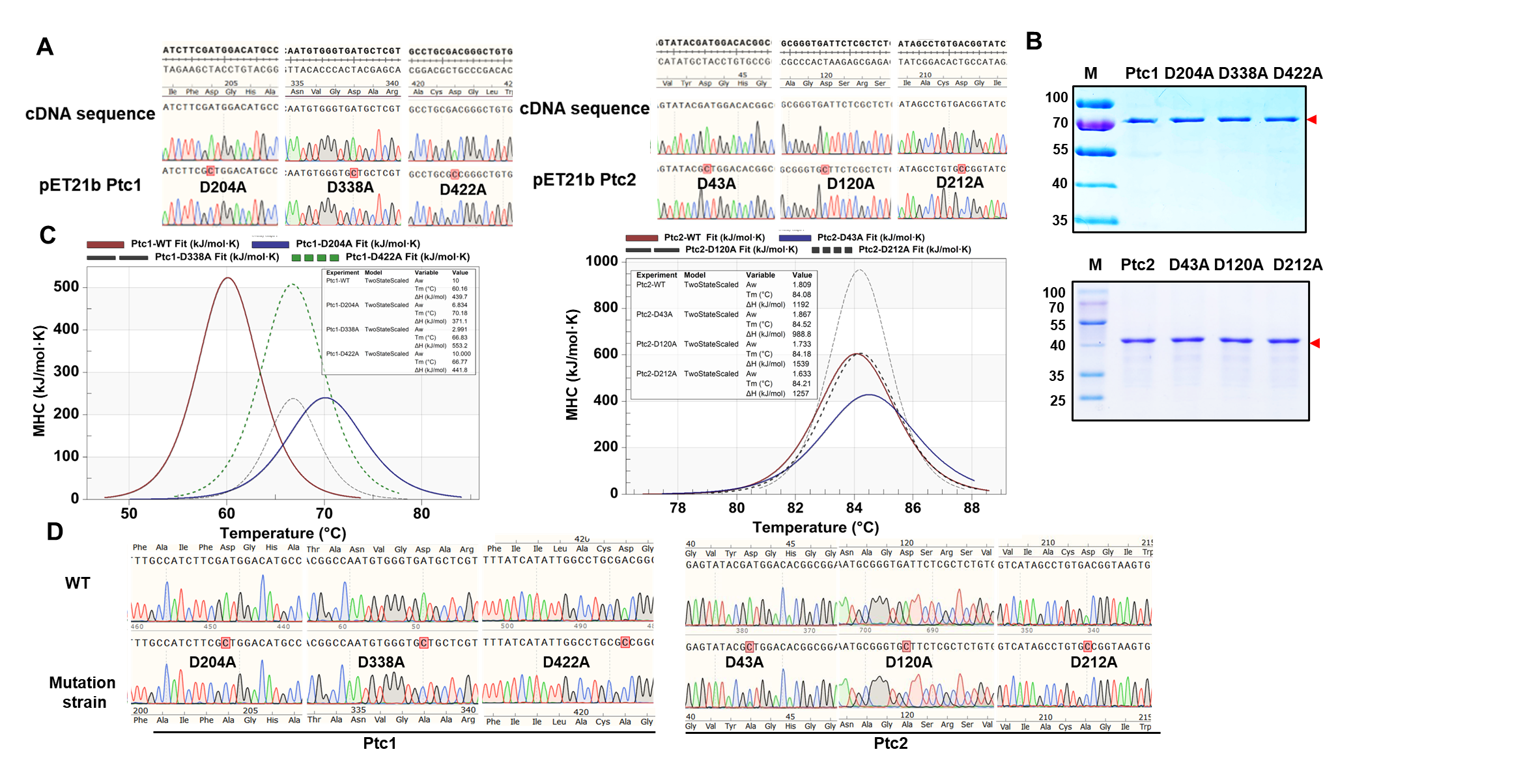
**

**Figure S9.** **Verification of Ptc1 and Ptc2 phosphatase point mutation proteins in *E. coli and A. flavus*** (A) Construction of phosphatase point mutation overexpression in *E. coli.* Verification of point mutation sequencing from *ptc1* and *ptc2* cDNA. (B) The inset shows purified Ptc1 and Ptc2 and their mutated versions in Coomassie Brilliant Blue-stained SDS-PAGE gel. His fusion proteins were expressed and purified in *E. coli.* (C) Thermal stability of Ptc1, Ptc2 and their mutant proteins *in* *vitro*. The purified protein samples were measured for the melting temperature using Nano differential scanning calorimetry (Nano-DSC) instrument; curve fitting and data analysis were performed in Nano-DSC Analysis System. (D) All site point mutations of *ptc1* and *ptc2* were further validated by gDNA sequencing in *A. flavus*.


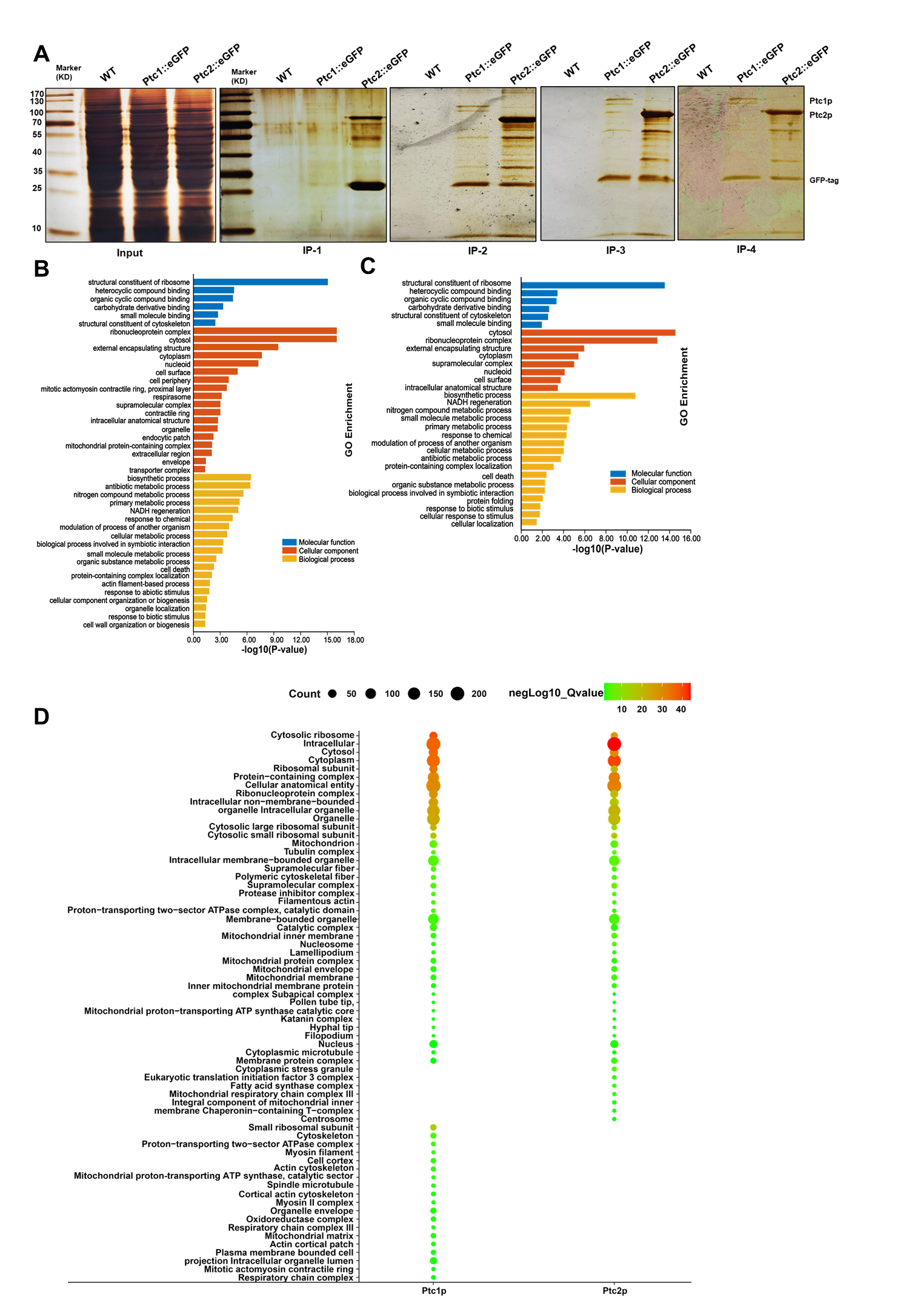


**Figure S10. Phosphatases Ptc1p and Ptc2p interact with proteins that regulate various biological processes in *A. flavus*.** (A) *A. flavus* samples expressing Ptc1::eGFP and Ptc2::eGFP were grown to aflatoxin-synthesis phase. WT were used as negative control. Protein lysate from WT, Ptc1::eGFP and Ptc2::eGFP strains immunoprecipitated by GFP nano-magnetic beads. The immunoprecipitates were subjected to SDS-PAGE followed by silver staining. The IP-MS experiment was repeated four times using the gel strip of IP-1 to 4 to complete lanes. (B-C) GO enrichment of all the identified proteins from Ptc1::eGFP and Ptc2::eGFP group. *P* values were determined using bootstrap sampling, with Benjamini-Hochberg correction. *P* ≤ 0.05 represent significantly enrichment results. (D) COMPARTMENTS enrichment of all identified proteins from Ptc1::eGFP and Ptc2::eGFP group. *P* values were determined *via* bootstrap sampling, with Benjamini-Hochberg correction. *Q* values (False discovery rate (FDR)–adjusted P) ≤ 0.05.


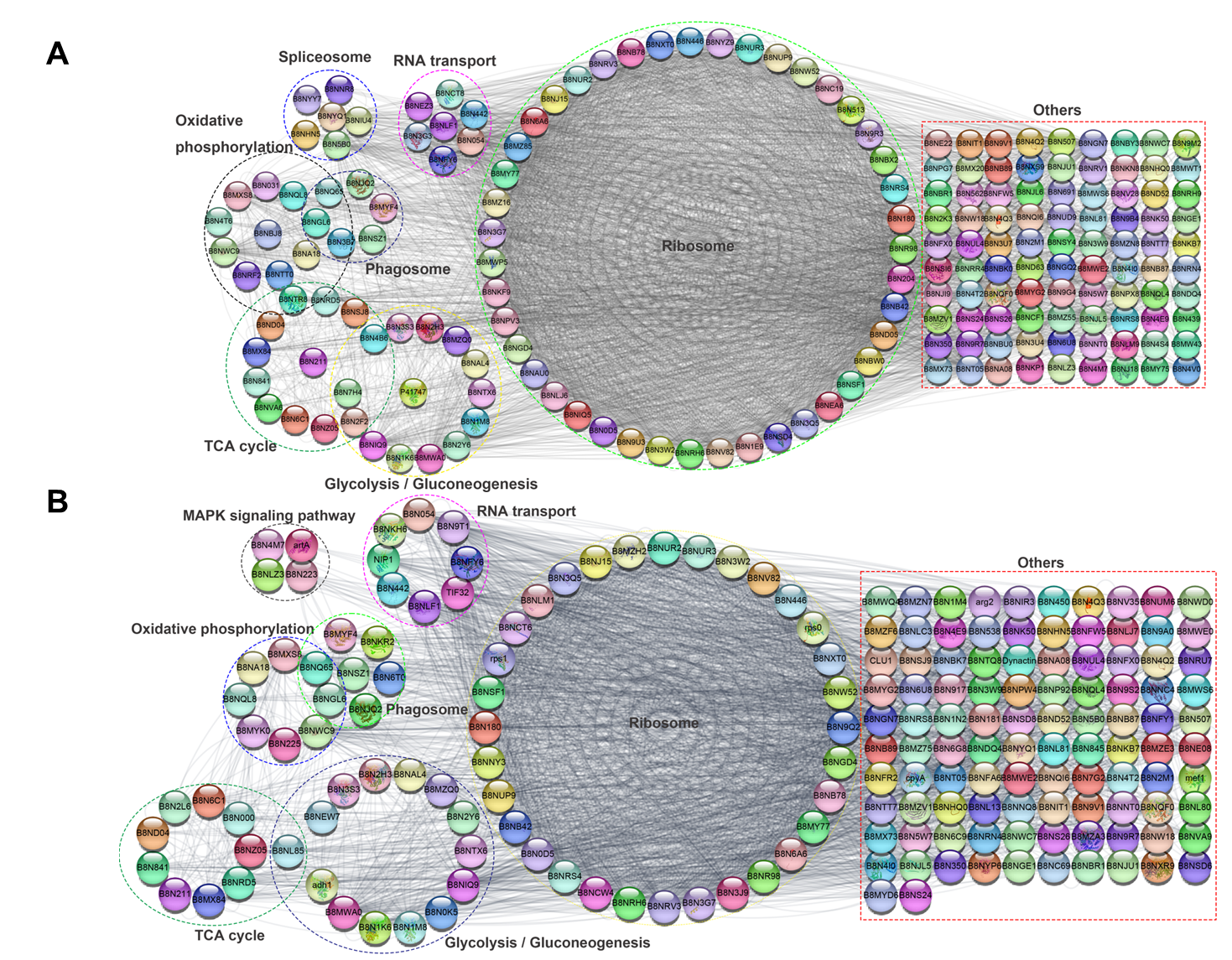


**Figure S11. Protein-protein interaction networks of all identified Ptc1- and Ptc2-interacting proteins.** (A-B) Protein-protein interaction (PPI) networks of 263 Ptc1-interacting proteins and 240 Ptc2-interacting proteins (contain Ptc1 or Ptc2 protein). By hiding disconnected nodes, the PPI network contained Ptc1-interacting and Ptc2-interacting protein under medium confidence (interaction score ≥ 0.4). The PPI network was generated from STRING database v11.5 and visualized in Cytoscape. The seven highly connected interaction clusters were generated by stringApp tool in Cytoscape


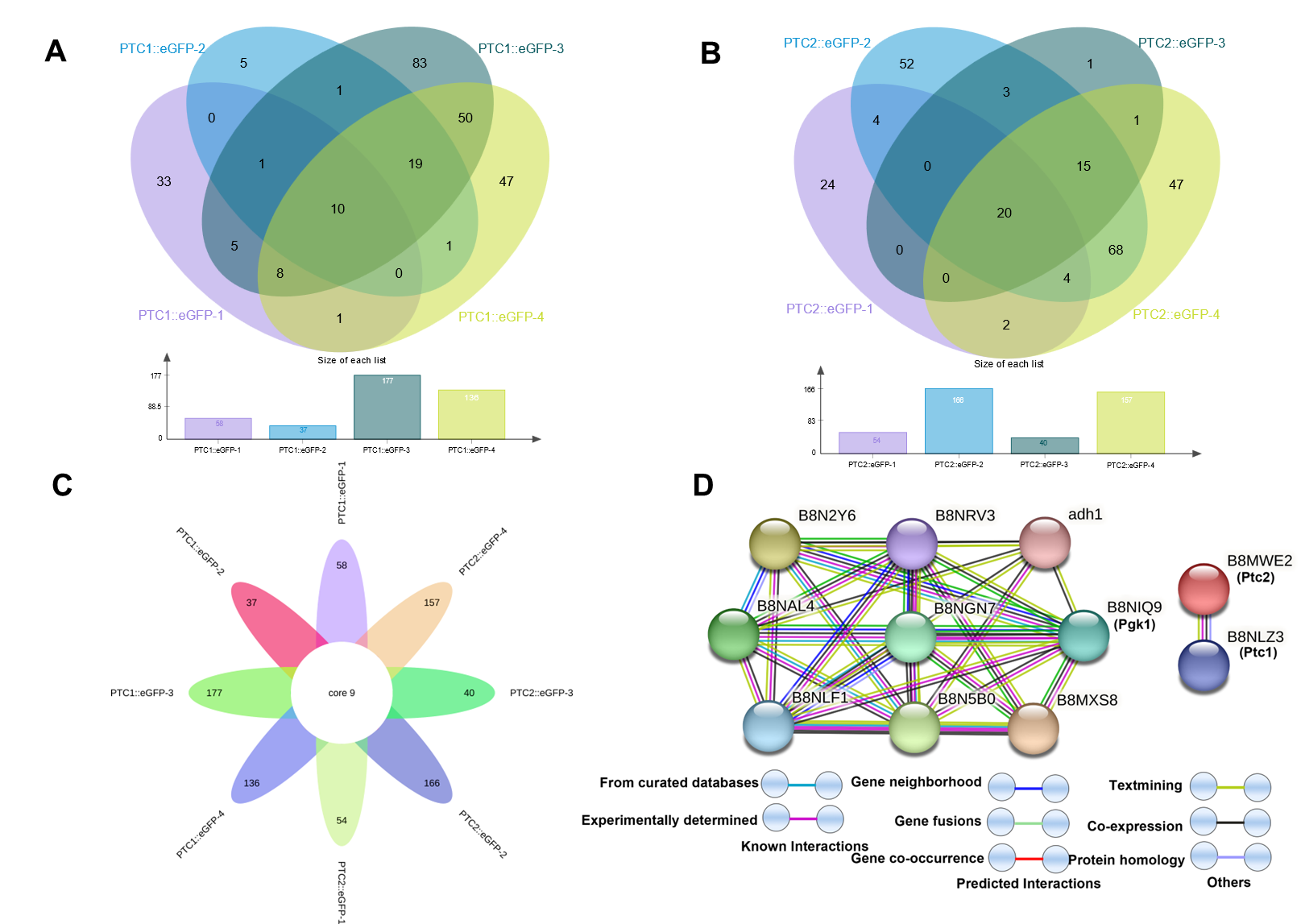


**Figure S12. Bioinformatics analysis of all identified Ptc1- and Ptc2-interacting proteins.** (A-B) Venn diagram showing the identified protein numbers of Ptc1::eGFP or Ptc2::eGFP group from four biological replicates. The bar chart below shows total number of proteins identified at each time of IP-MS experiment. (C) Flower diagram showing the identified protein numbers of Ptc1::eGFP or Ptc2::eGFP group from four biological replicates of IP-MS data. The flower core shows 9 overlapping interacting proteins of both Ptc1 and Ptc2 in each experiment. (D) Protein-protein interaction networks of 9 overlap interacting proteins of both Ptc1 and Ptc2 (contains Ptc1 and Ptc2 proteins; interaction score ≥ 0.4).


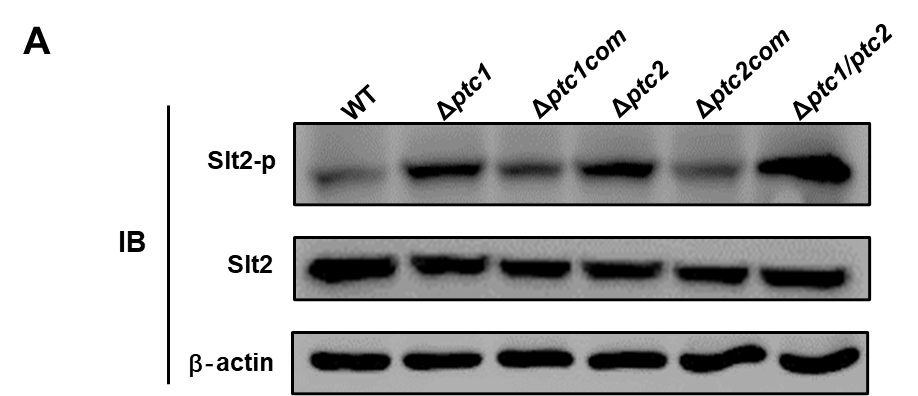


**Figure S13. Ptc1 and Ptc2 are involved in dephosphorylation of Slt2 *in* *vivo*.** (A) Protein extracts were prepared, and the levels of phosphorylated Slt2 were detected in WT, Δ*ptc1*, Δ*ptc1*com, Δ*ptc2*, Δ*ptc2*com, and Δ*ptc1*/Δ*ptc2* strains by immunoblotting withanti-P-Slt2/anti-Slt2 antibody. β-actin was used as control and detected using an anti-actin antibody. The intensity of fusion protein in WT was defined as 100.


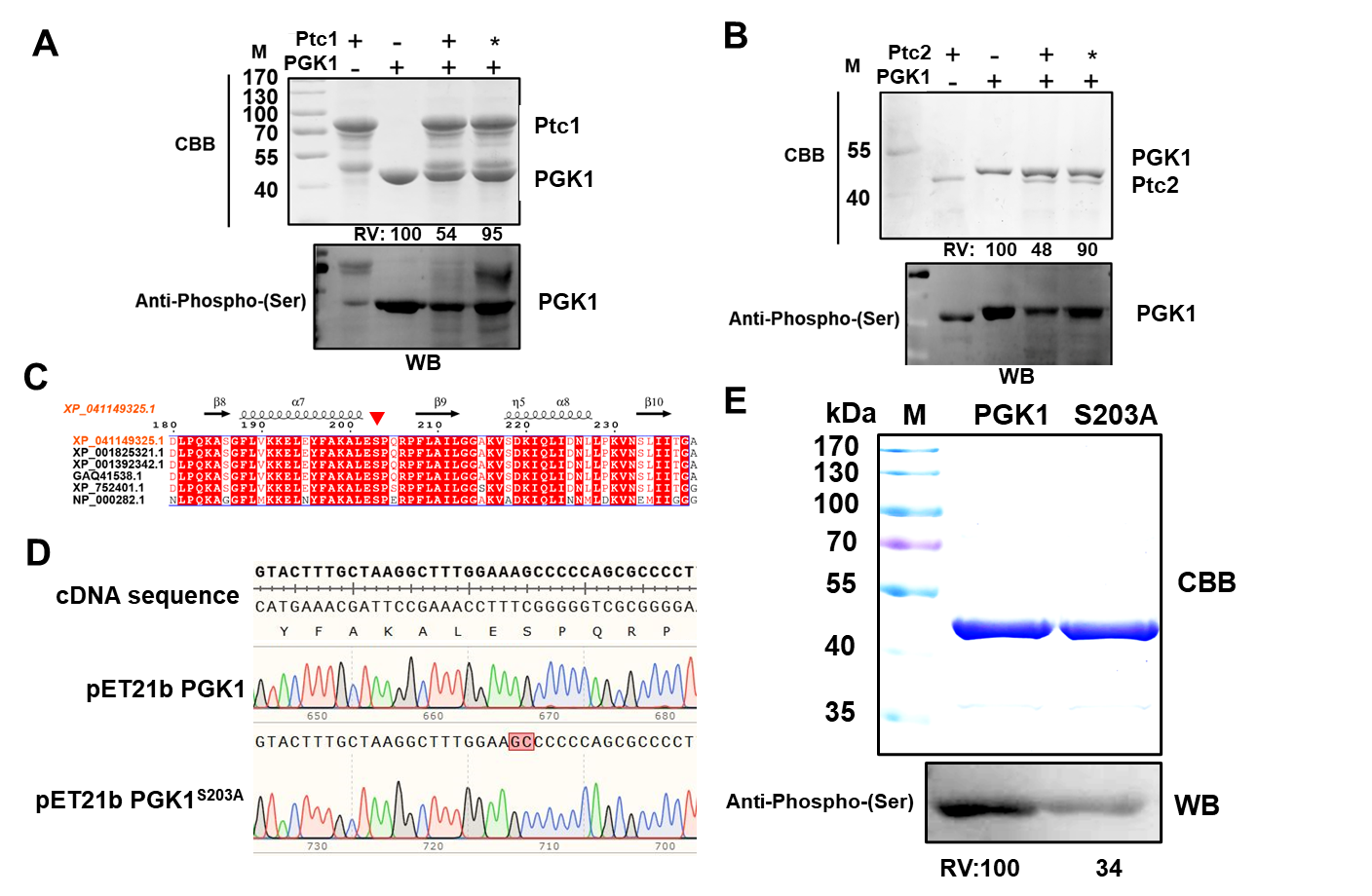


**Figure S14. Ptc1 and Ptc2 can catalyze PGK1 Ser dephosphorylation in *vitro.*** (A–B) Coomassie Brilliant Blue-stained SDS-PAGE of purified PGK1, Ptc1, and Ptc2 from *E. coli*. PGK1 was combined with Ptc1/Ptc2 or heat-inactivated Ptc1/Ptc2(*) *in vitro* at 37℃ for 30 min and underwent a western blotting analysis with anti-phospho-(Ser). CBB is defined as Coomassie brilliant blue. (C) Structure-based sequence alignment of PGK1 with NCBI BLAST and ESPript 3.0 (http://espript.ibcp.fr/ESPript/ESPript/). Identical residues among all proteins are shown in white letters over a red background. Secondary structural elements are indicated as α, β, and η. (D) Construction of PGK1^S203A^ point mutation overexpression proteins in *E. coli.* Verification of point mutation sequencing was carried out from PGK1 and PGK1^S203A^ cDNA. (E) Effects of S203A mutations on the serine phosphorylation of PGK1 *in* *vitro.* Coomassie Brilliant Blue-stained SDS-PAGE of purified PGK1 and PGK1 mutants (S203A). A western blotting analysis was used to detect the protein serine phosphorylation levels with anti-phospho-ser antibody. PGK1 served as positive control. CBB is defined as Coomassie brilliant blue.

*
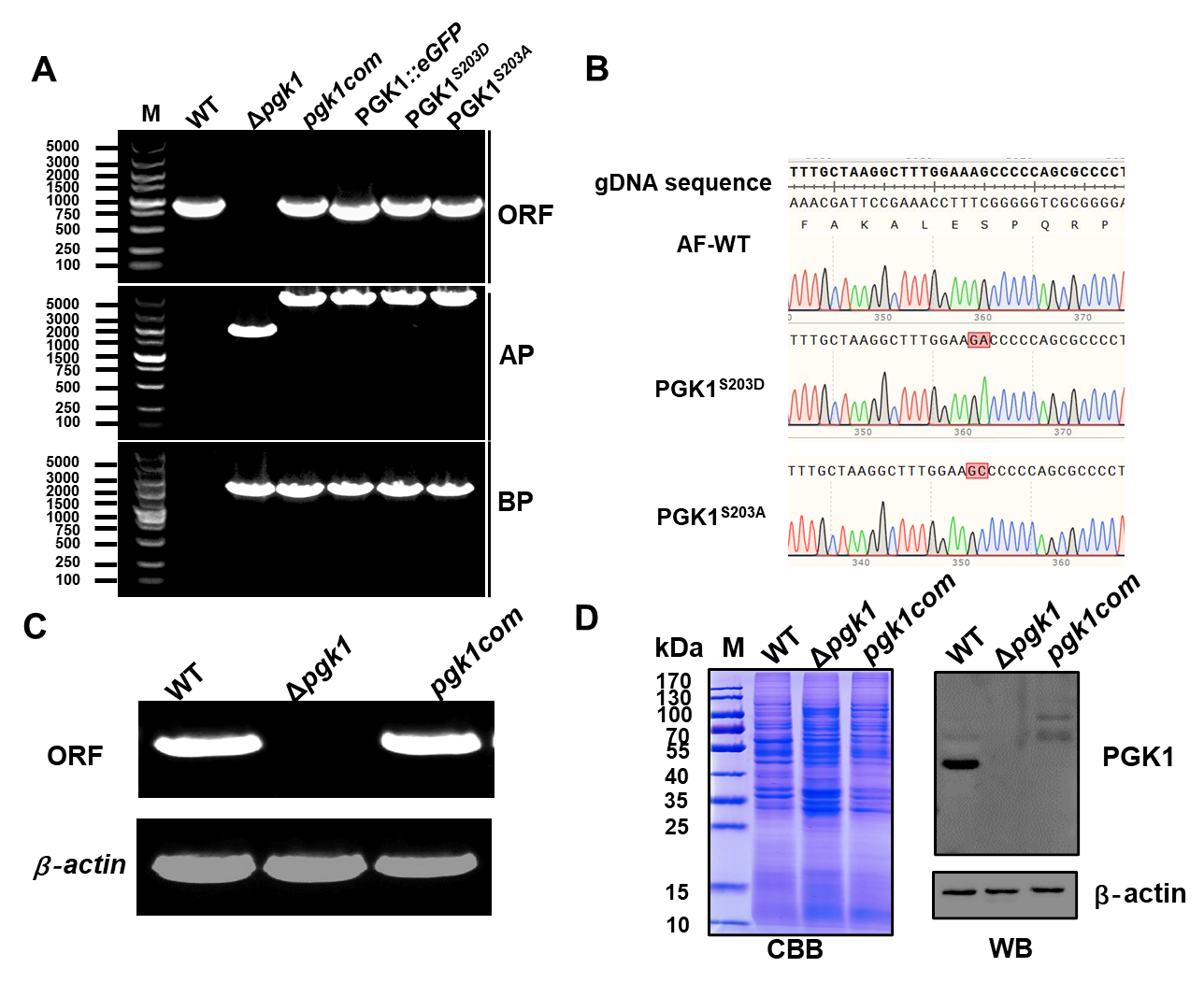
*

**Figure S15.** **Construction of PGK1 knockout mutant, site point mutant, and GFP fusion protein strain using homologous recombination.** (A) Knockout, complement, site point mutation, and fusion protein strains of PGK1 were validated by PCR in *A. flavus.* AP represents the upstream region of the strain detected using *pgk1* AF and P-801R primers; BP represents the downstream region of the strain detected using *pgk1* BR and P-1020F primers; ORF region was detected using ORF detection primers (*pgk1*-D-F/R) (B) All site point mutations of *pgk1* were validated by gDNA sequencing in *A. flavus*. (C) The knockout verification of genes (panel A) was performed using RT-PCR with their RNA in *A. flavus* and with *β-actin* as a control. (D) Knockout verification of WT, Δ*pgk1*, and *pgk1*com by western blotting using their proteins in *A. flavus.* The proteins separated by SDS-PAGE were visualized by staining gel with Coomassie Brilliant Blue (CBB) (left) or by transferring samples to a polyvinylidene fluoride membrane, followed by immunoblotting. The primary antibody was anti-PGK1 and β-actin was loading control in detection of PGK1 protein.


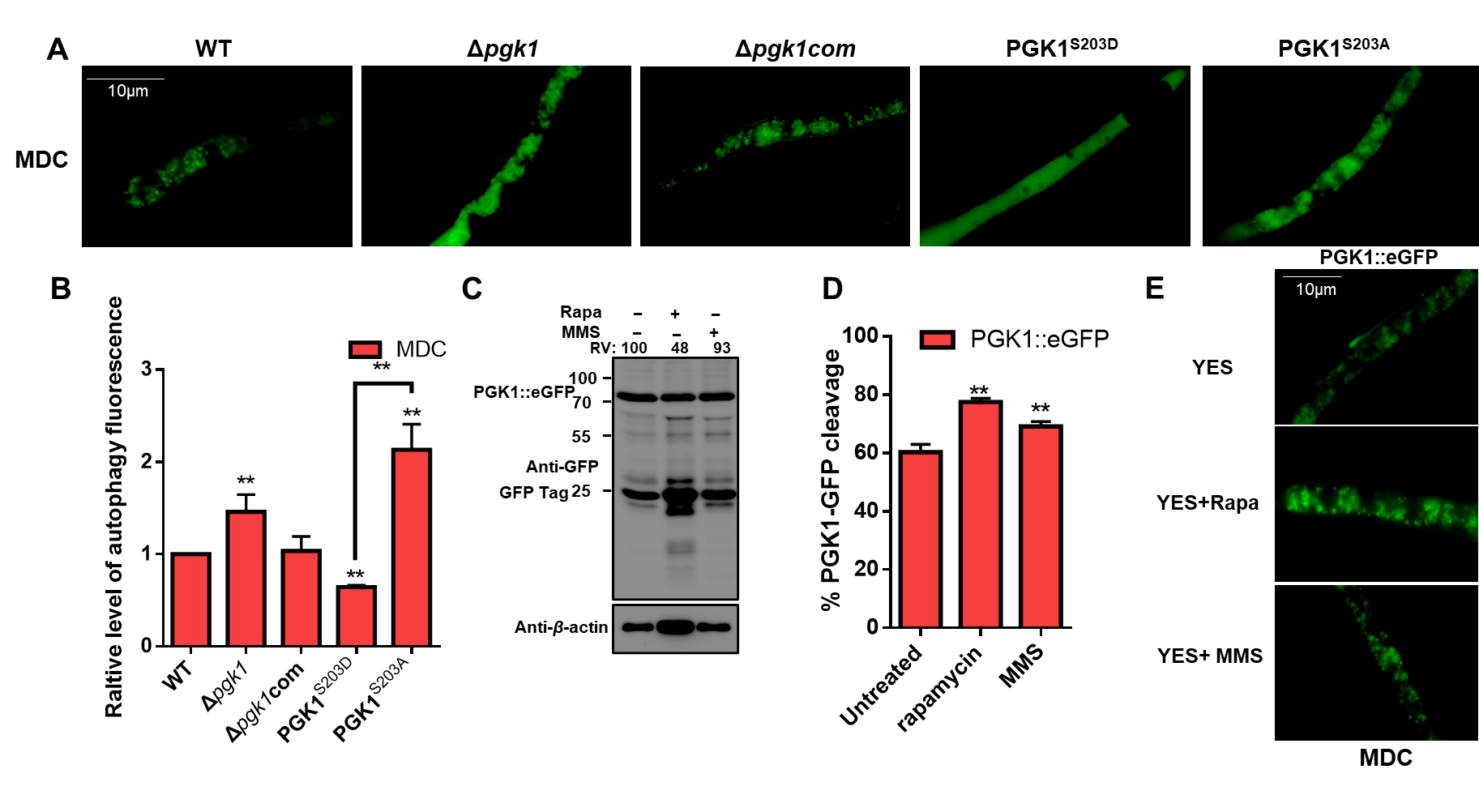


**Figure S16. Role of PGK1 in formation of autophagic vesicles in *A. flavus.*** (A) Fluorescent microscopic imaging analysis of autophagic fluorescence was carried out in the WT, Δ*pgk1*, Δ*pgk1*com, *pgk1*^S203D^, and *pgk1*^S203A^ stained by MDC. (B) MDC staining for autophagic vesicle detection. The fluorescence intensity of MDC stain in WT, Δ*pgk1*, Δ*pgk1*com, *pgk1*^S203D^, and *pgk1*^S203A^ strains was determined using SpectraMax i3x. Error bars represent SD from 3 independent experiments. Statistical analyses were performed by a one-way ANOVA followed by Dunnett-T post-hoc test. (***P* ≤ 0.01). (C) Immunoblotting analysis showing processing of PGK1::eGFP fusion proteins in PGK1::eGFP strains after rapamycin or MMS treatments. Total proteins were extracted and analyzed with anti-GFP antibodies. Actin was used as protein loading control. The intensity of fusion protein in untreated group was defined as 100. Values below each lane represent expression level of PGK1 protein as quantified by ImageJ. (D) The bar chart represents ratio of free GFP to total GFP signal in the lane as described in Fig. S16C. Error bars represent SD from 3 independent experiments. Statistical analyses were performed by a one-way ANOVA followed by Dunnett-T post-hoc test. (***P* ≤ 0.01). (E) Autophagic vesicles were determined by MDC staining in PGK1::eGFP strains after rapamycin or MMS treatments (scale bar, 10 μm).

Table S1. Wild-type, mutant strains and other strains used in this study.

Table S2. Primers used for qRT-PCR, mutants and recombinant vector construction

Table S3. Complete list of proteins identified in IP-MS experiments.

Table S4. eggNOG-mapper protein annotations for identified proteins.

Table S5. List of Ptc1 and Ptc2 interacting proteins identified in all four biological replicates of IP-MS.

Table S6. Subcellular localization of identified proteins.

Table S7. GO function enrichment analysis of identified proteins.

Table S8. KEGG PATHWAT and COMPARTMENTS enrichment analysis of identified proteins.

Table S9. PPI for identifying proteins from STRING database.
